# Supplementary material for: Following the Fate of Lytic Polysaccharide Monooxygenases under Oxidative Conditions by NMR Spectroscopy
Source: Biochemistry. 2023 May 31;62(12):1976–93. doi: 10.1021/acs.biochem.3c00089 (PMC10286315; doi:10.1021/acs.biochem.3c00089)
Supplement: Supplementary file 1 — bi3c00089_si_001.pdf [file bi3c00089_si_001.pdf]

Supporting information

Following the fate of lytic polysaccharide monooxygenases (LPMOs) under oxidative conditions by NMR spectroscopy

Idd A. Christensen[1], Vincent G. H. Eijsink[2,*], Anton A. Stepnov[2], Gaston Courtade[1], Finn L. Aachmann[1,*].

[1]NOBIPOL, Department of Biotechnology and Food Science, NTNU Norwegian University of Science and Technology, Sem Sælands vei 6/8, 7491 Trondheim, Norway

[2]Faculty of Chemistry, Biotechnology and Food Science, NMBU - Norwegian University of Life Sciences, 1432 Ås, Norway

ORCIDs

Idd A. Christensen idd.a.christensen@ntnu.no: 0000-0001-6229-2211

Vincent G. H. Eijsink vincent.eijsink@nmbu.no: 0000-0002-9220-8743

Anton Stepnov anton.stepnov@nmbu.no: 0000-0002-6754-5852

Gaston Courtade gaston.courtade@ntnu.no: 0000-0002-1644-3223

Finn L. Aachmann finn.l.aachmann@ntnu.no: 0000-0003-1613-4663

[*] **To whom correspondence should be addressed.**
Finn L. Aachmann
NOBIPOL, Department of Biotechnology and Food Science, NTNU Norwegian University of Science and Technology, Sem Sælands vei 6/8, N-7491 Trondheim, Norway
Tel.: +47 97954859

Vincent G. H. Eijsink
Faculty of Chemistry, Biotechnology and Food Science, NMBU - Norwegian University of Life Sciences, 1432 Ås, Norway
Tel: +47 67232463

E-mail: finn.l.aachmann@ntnu.no, vincent.eijsink@nmbu.no

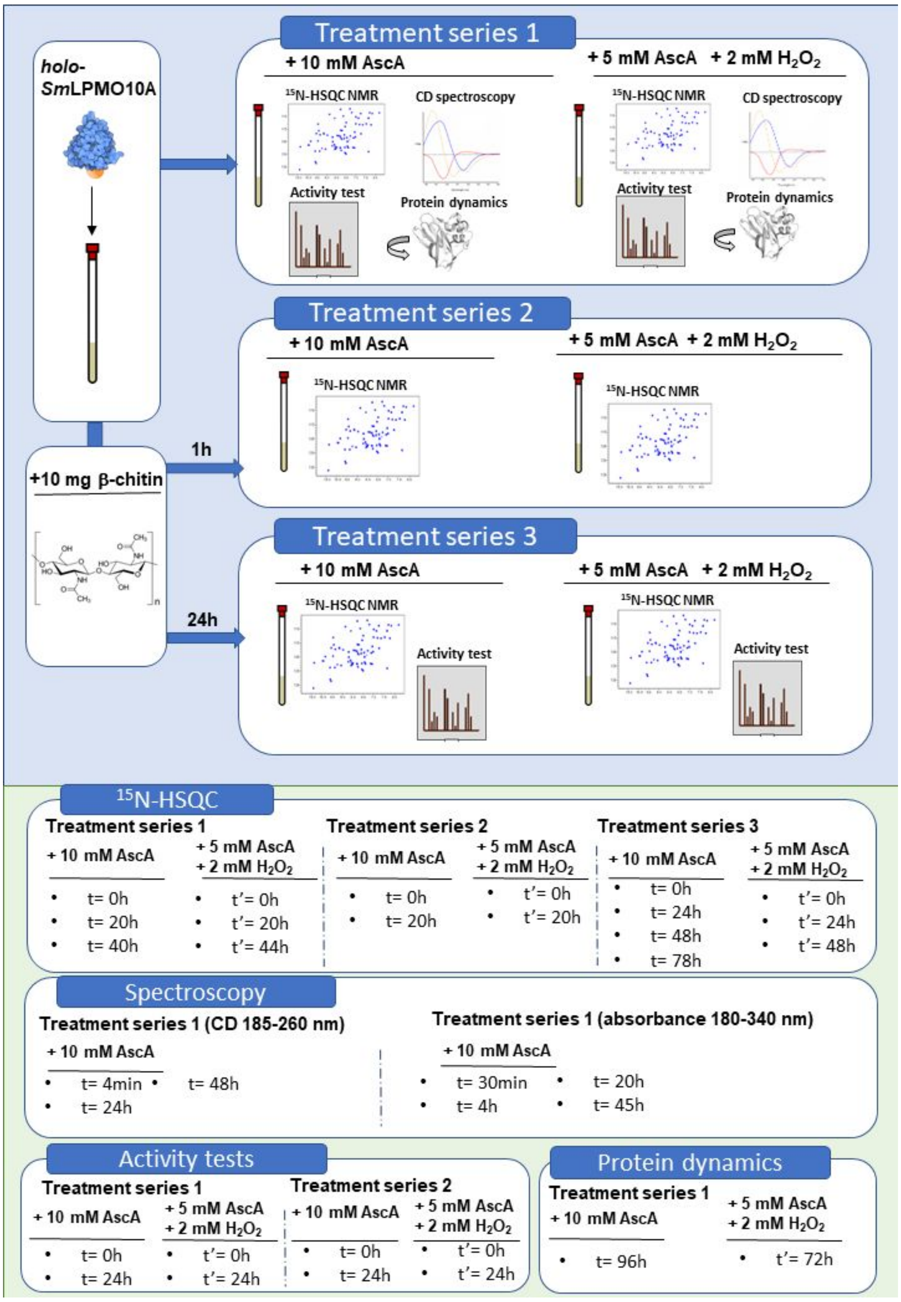

Figure S1: Overview of the experimental work done to observe the structural effects of oxidative conditions on *holo*-*Sm*LPMO10A. The effects of oxidative conditions were followed in three treatment series. Treatment series 1 was carried out in the absence of the substrate chitin, while *holo*-*Sm*LPMO10A was pre-incubated with 10 mg β-chitin particles for 1h and 24h in treatment series 2 and 3, respectively. Oxidative conditions were induced in all three treatment series by, first, the addition of 10 mM ascorbic acid and, later, the addition of 5 mM ascorbic acid and 2 mM H_2O_2. The methods used to monitor the effects of the oxidative conditions are indicated by icons for each treatment series, and the time-points for the various measurements are provided for each treatment series in the panels below. All samples were prepared in acetate buffer (25 mM sodium-acetate, 25 mM acetic acid, 10 mM NaCl, pH 5.0) with ~ 100-150 µM *holo*-*Sm*LPMO10A. Individual samples were prepared for each of the methods used in each treatment series.

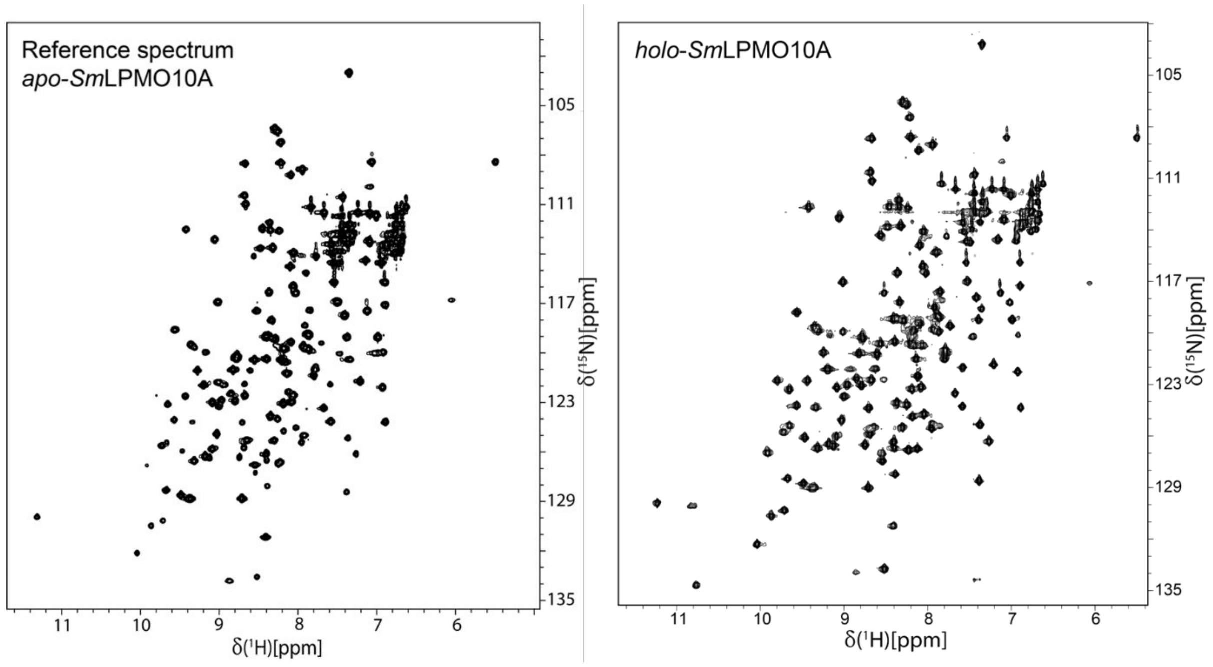

Figure S2: ^{15}N-HSQC spectra of *apo*-*Sm*LPMO10A and *holo*-*Sm*LPMO10A in acetate buffer (25 mM sodium-acetate, 10 mM NaCl, pH 5.0). The ^{1}H-^{15}N-signals in the spectrum are narrow and dispersed in both dimensions indicating that *Sm*LPMO10A is correctly folded. In the case of *holo*-*Sm*LPMO10A ^{1}H-^{15}N signals of residues within ~10Å from the copper active site relax too fast to be detected in the spectrum, due to Paramagnetic Relaxation Enhancement caused by binding of Cu(II) to the active site.

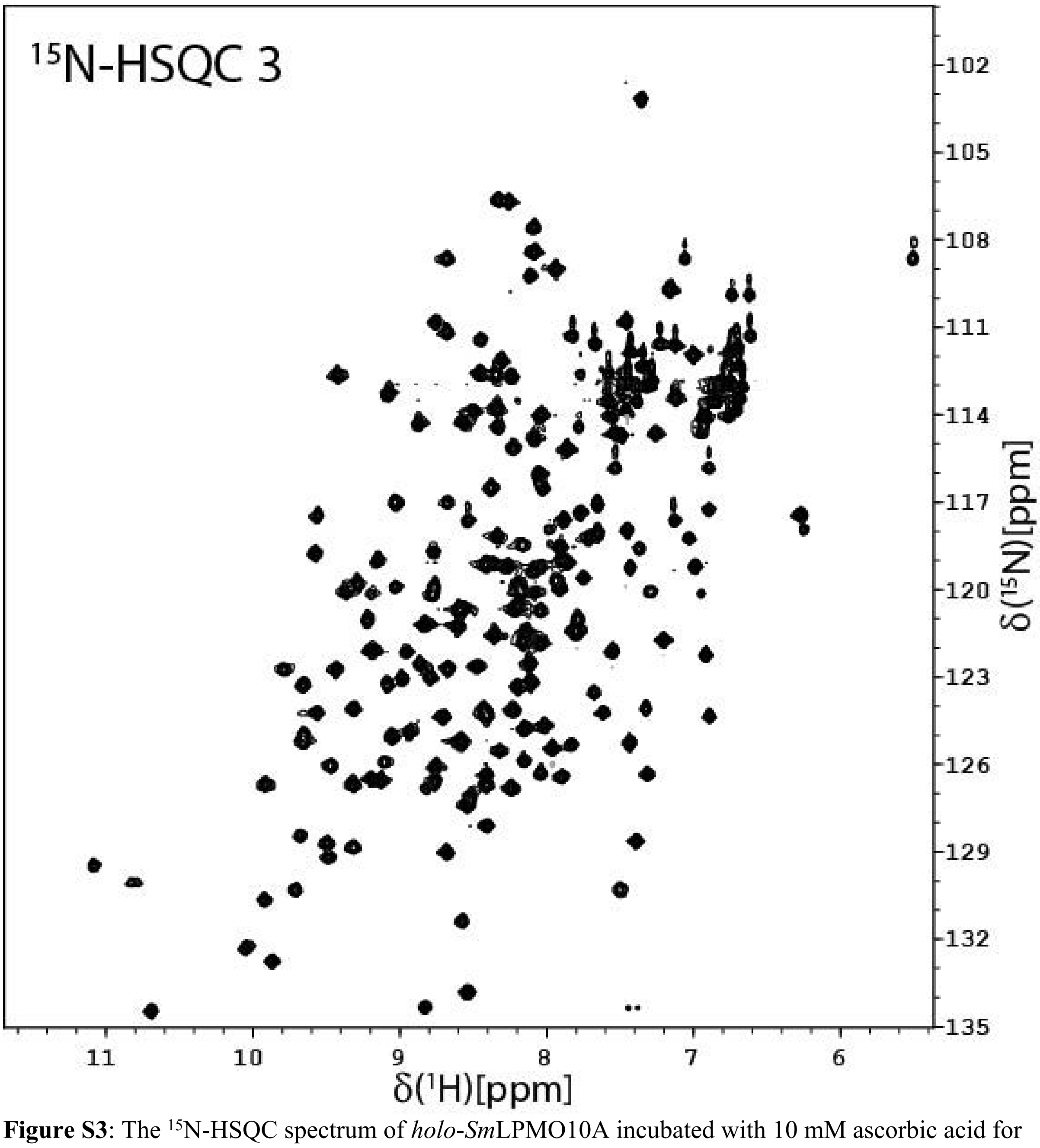

Figure S3: The ^{15}N-HSQC spectrum of *holo-Sm*LPMO10A incubated with 10 mM ascorbic acid for t= 0h in acetate buffer (25 mM sodium-acetate, 10 mM NaCl, pH 5.0) at 25 °C. (treatment series 1; ^{15}N-HSQC 3)

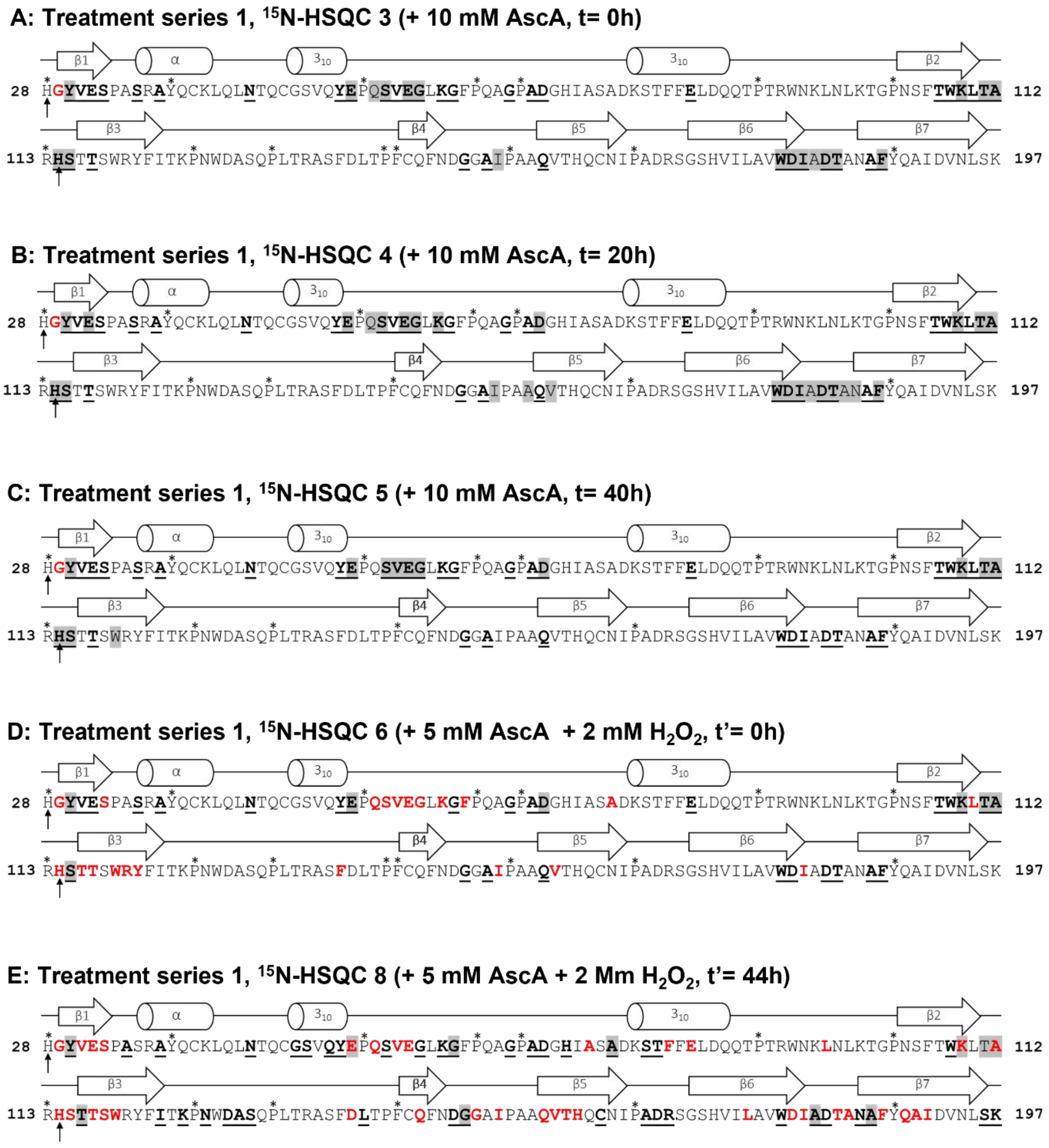

Figure S4: The combined structural changes to *holo*-*Sm*LPMO10A observed in treatment series 1, relative to the reference spectrum of *apo*-*Sm*LPMO10A. The active site histidines are indicated by arrows, while residues not assigned in by NMR are indicated by asterixis. Chemical shift perturbations >50Hz are shown as by bold and underlined residues, narrower linewidths (i.e increased signal intensities) are shown by residues highlighted in grey. Non-detectable ^{1}H-^{15}N signals are shown by red residues.

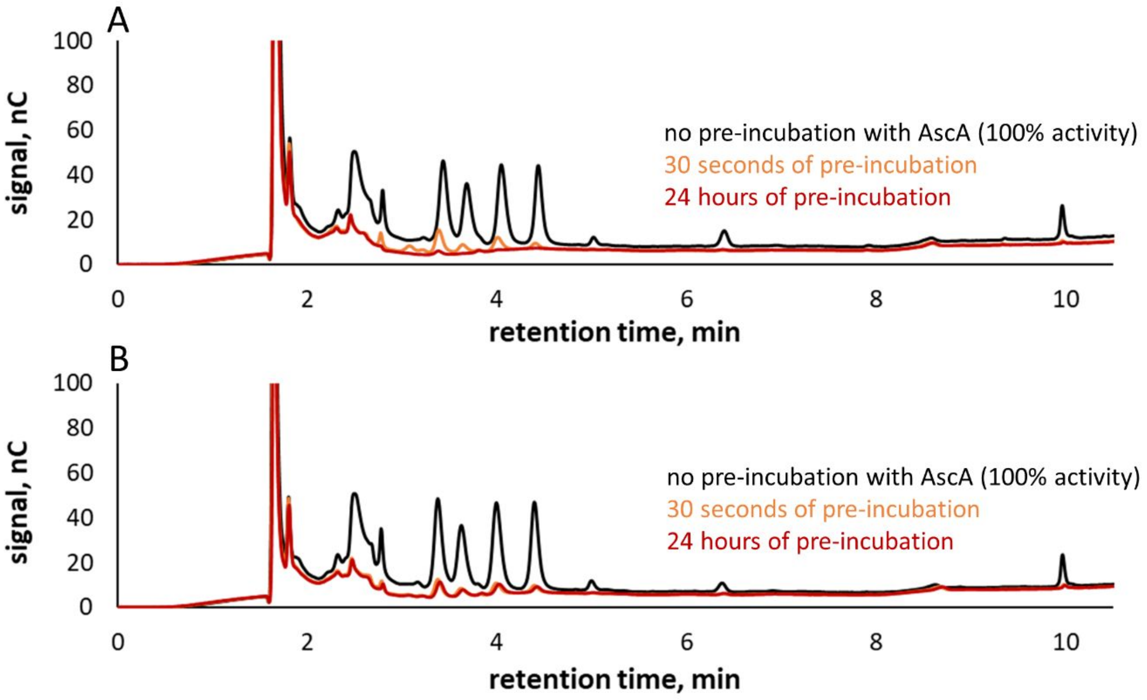

Fig. S5. Qualitative analysis of oxidative inactivation of *Sm*LPMO10A in the presence of ascorbic acid. The figure shows HPAEC-PAD chromatograms of LPMO reaction products obtained by treating β-chitin with S*m*LPMO10A that had been pre-incubated with ascorbic acid in the absence (A) or presence (B) of ≈2% (w/w) β-chitin. The pre-incubation experiments were carried out at room temperature using 100 µM LPMO and 10 mM ascorbic acid in acetate buffer (25 mM sodium acetate, 10 mM NaCl, pH 5.0). After pre-incubation and filtration, the enzyme solutions were diluted 100-fold in sodium acetate buffer, pH 5.0, containing ≈1% (w/w) β-chitin and 1 mM ascorbic acid, and further incubated for 24 h at 40 °C to assess the residual activity. Note that chromatograms of reaction products obtained in residual activity experiments (orange and red traces) indicate considerable reductions in enzyme activity compared to a reference reaction with the LPMO not subjected to pre-incubation with a reductant (black trace). A key difference between the upper and lower panel is that in the lower panel (pre-incubation with b-chitin), the enzyme still shows activity after 24 h of pre-incubation, as discussed in more detail in the main text. To obtain quantitative data on enzyme inactivation, the LPMO reaction products were treated with *Sm*GH20 chitobiase converting them to a mixture of *N*-acetylglucosamine (GlcNAc) and chitobionic acid (GlcNAcGlcNAc1A), and the amount of chitobionic acid (i.e., the total amount of soluble oxidized products) was determined by HPAEC-PAD. Such chitobionic acid levels were used to produce Figure 4 in the main text.

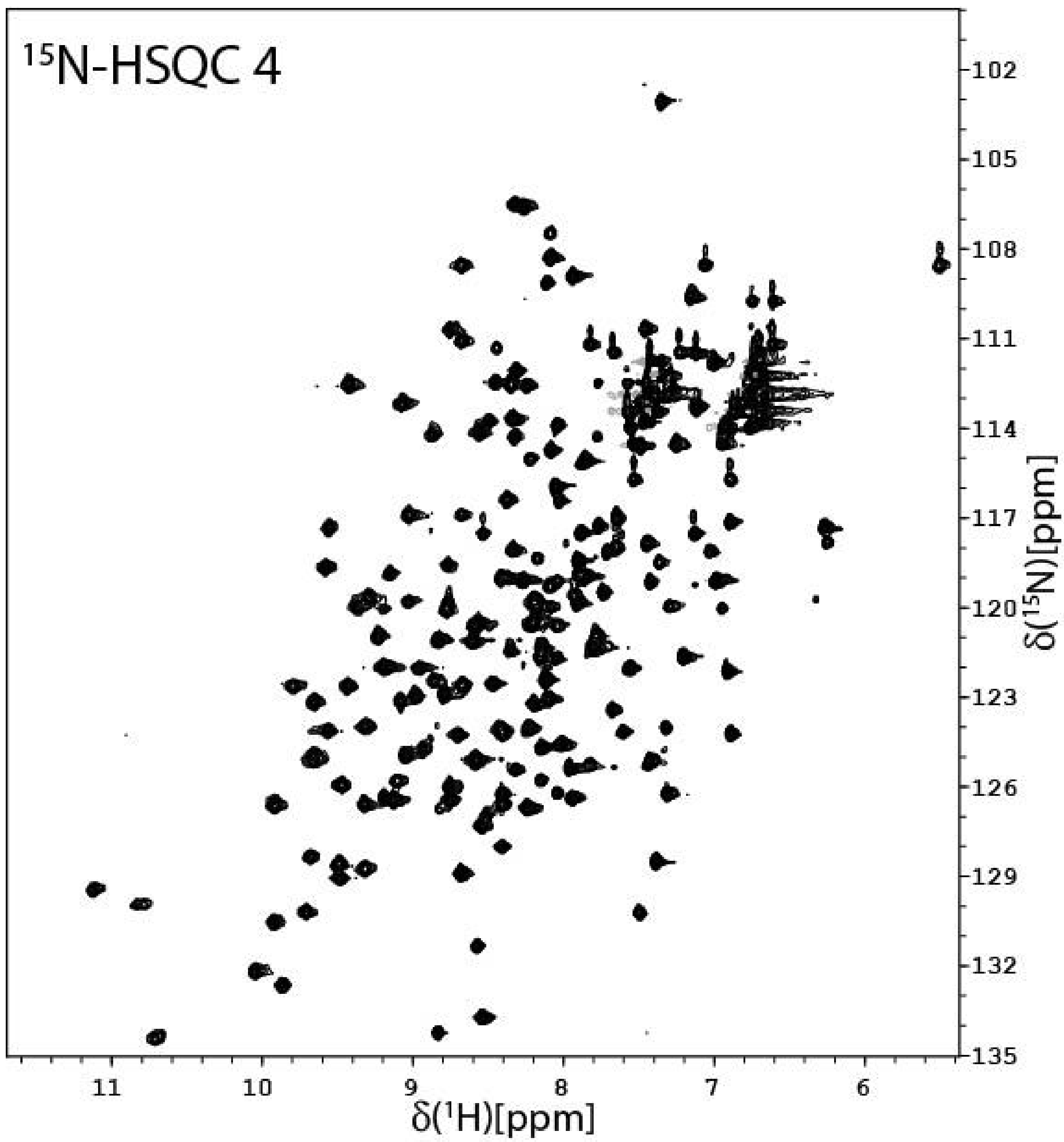

Figure S6: The ^{15}N-HSQC spectrum of *holo*-*Sm*LPMO10A incubated with 10 mM ascorbic acid for t= 20h in acetate buffer (25 mM sodium-acetate, 10 mM NaCl, pH 5.0) at 25 °C (treatment series 1; ^{15}N-HSQC 4).

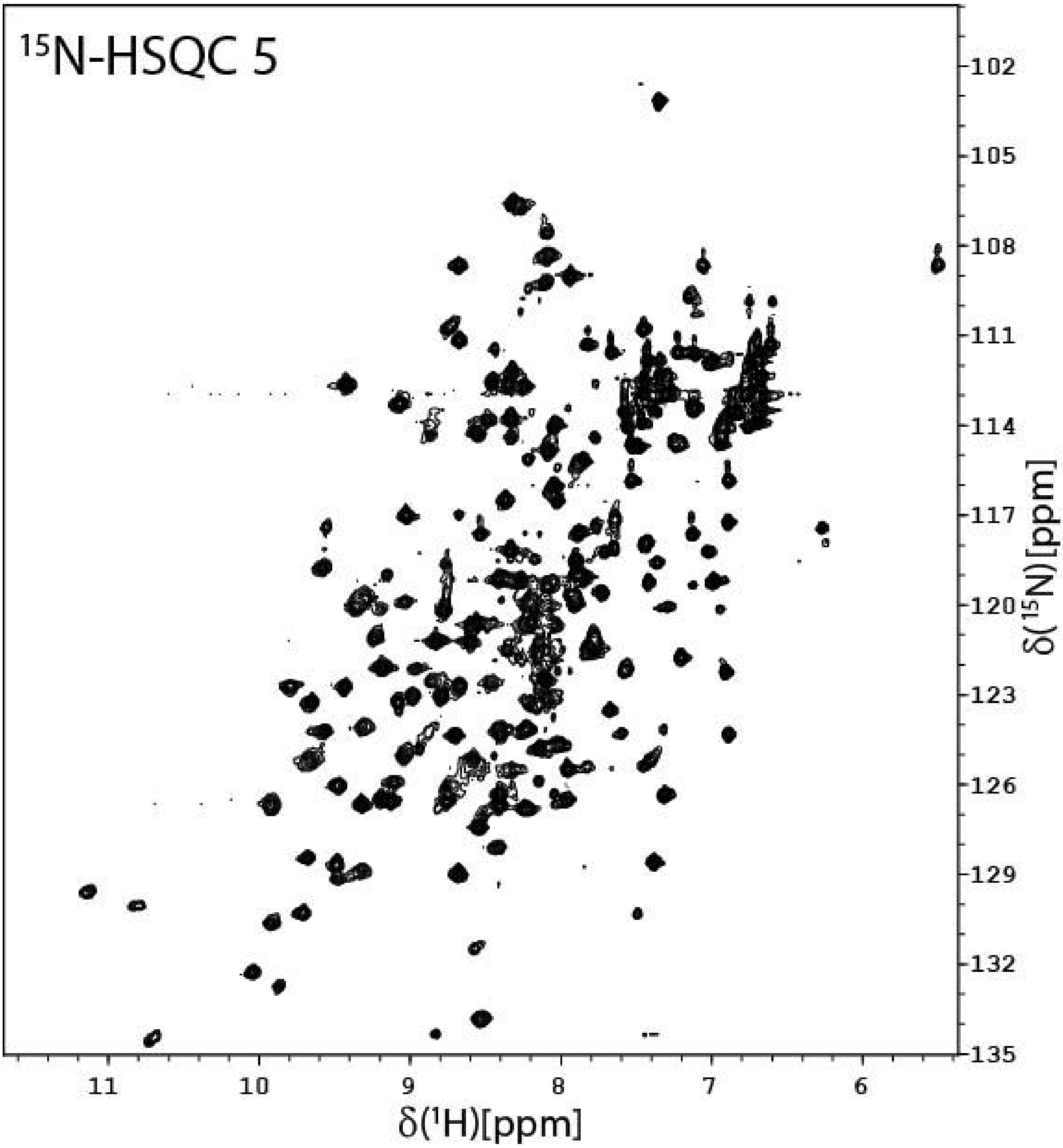

Figure S7: The ^{15}N-HSQC spectrum of *holo*-*Sm*LPMO10A incubated with ascorbic acid for t= 40h in acetate buffer (25 mM sodium-acetate, 10 mM NaCl, pH 5.0) at 25 °C. (treatment series 1; ^{15}N-HSQC 5).

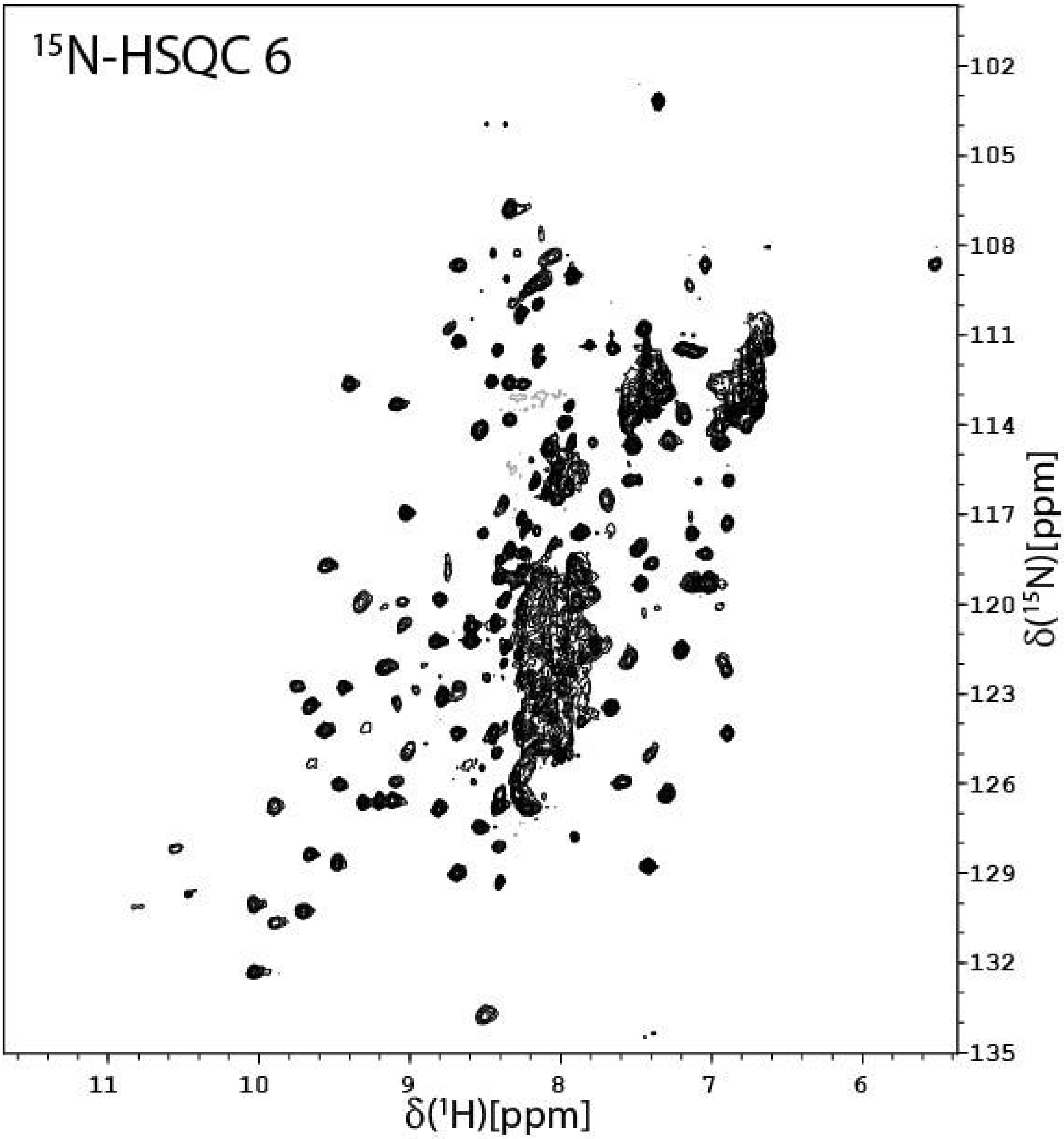

Figure S8: The ^{15}N-HSQC spectrum of *holo-Sm*LPMO10A incubated with 10 mM ascorbic acid for 40h and, subsequently, with 5 mM ascorbic acid and 2 mM H_2O_2 for t'= 0h in acetate buffer (25 sodium-acetate, 10 mM NaCl, pH 5.0) at 25 °C (treatment series 1; ^{15}N-HSQC 6).

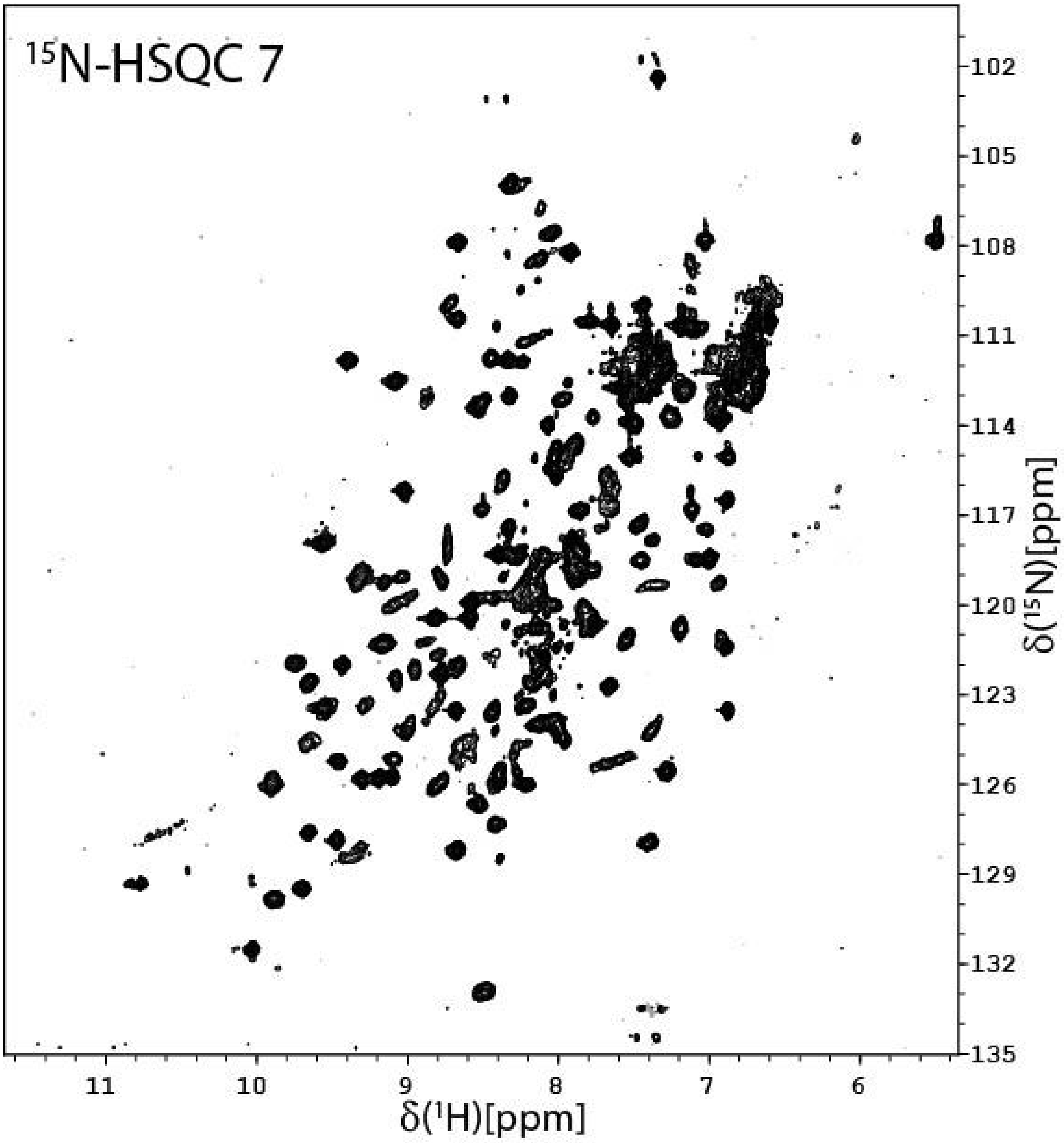

Figure S9: The ^{15}N-HSQC spectrum of *holo*-*Sm*LPMO10A incubated with 10 mM ascorbic acid for 40h and, subsequently, with 5 mM ascorbic acid and 2 mM H_2O_2 for t'= 20h in acetate buffer (25 mM sodium-acetate, 10 mM NaCl, pH 5.0) at 25 °C (treatment series 1; ^{15}N-HSQC 7).

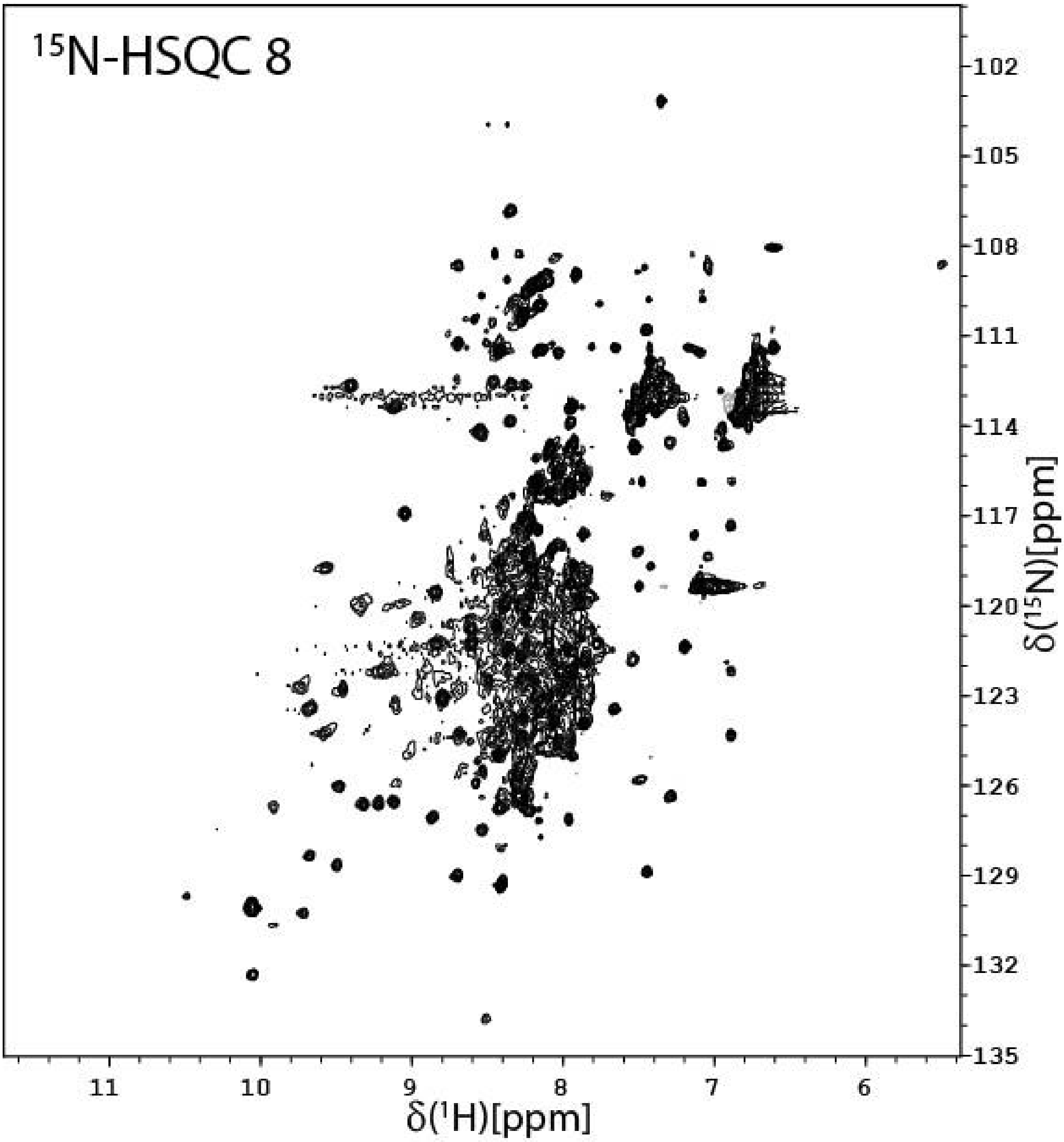

Figure S10: The ^{15}N-HSQC spectrum of *holo*-*Sm*LPMO10A incubated with 10 mM ascorbic acid for 40h and, subsequently, with 5 mM ascorbic acid and 2 mM H_2O_2 for t'= 44h in acetate buffer (25 mM sodium-acetate, 10 mM NaCl, pH 5.0) at 25 °C (treatment series 1; ^{15}N-HSQC 8).

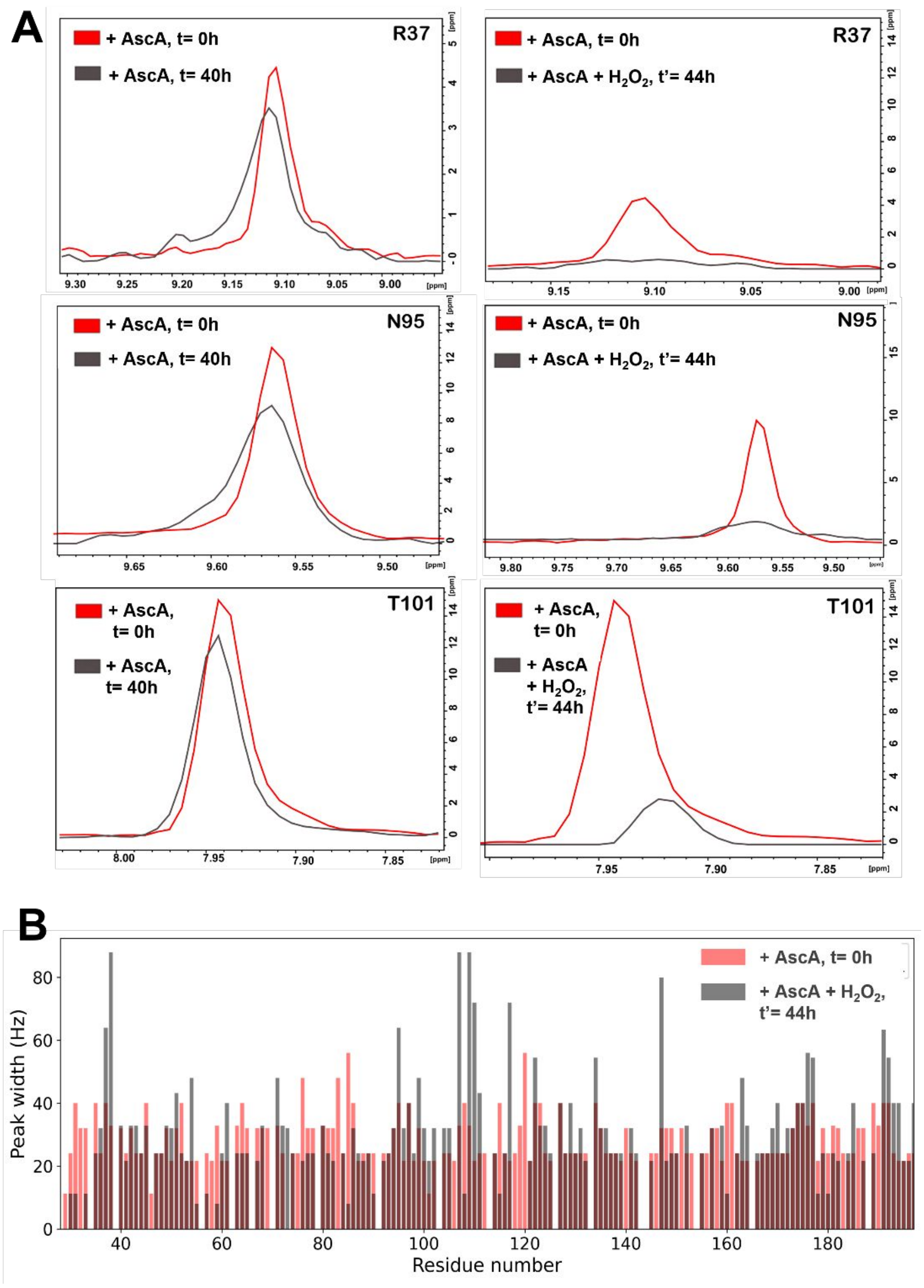

Figure S11: Changes in the ^{1}H resonance linewidths of *Sm*LPMO10A in response to incubation with ascorbic acid and, subsequently, ascorbic acid and H_2O_2, in the absence of chitin. **(A)** Changes in the ^{1}H resonances of residues R37, N95, and T101 in response to incubation with ascorbic acid alone (t= 0h or t= 40h), and, subsequently, ascorbic acid together with H_2O_2 (t'= 44h). The t= 0 timepoint is after the addition of ascorbic acid until t= 40min, which is the acquisition time for the ^{15}N-HSQC spectrum. The linewidths of R37 and N95 increase in response to the treatments, while the linewidth of T101 remains unchanged; addition of H_2O_2 next to ascorbic acid speeds up line widening. **(B)** Changes in the ^{1}H resonance linewidth of all residues of *Sm*LPMO10A in response to incubation with 10 mM ascorbic acid (t= 40h) and, subsequently, with 5 mM ascorbic acid and 2 mM H_2O_2 (t'= 44h).

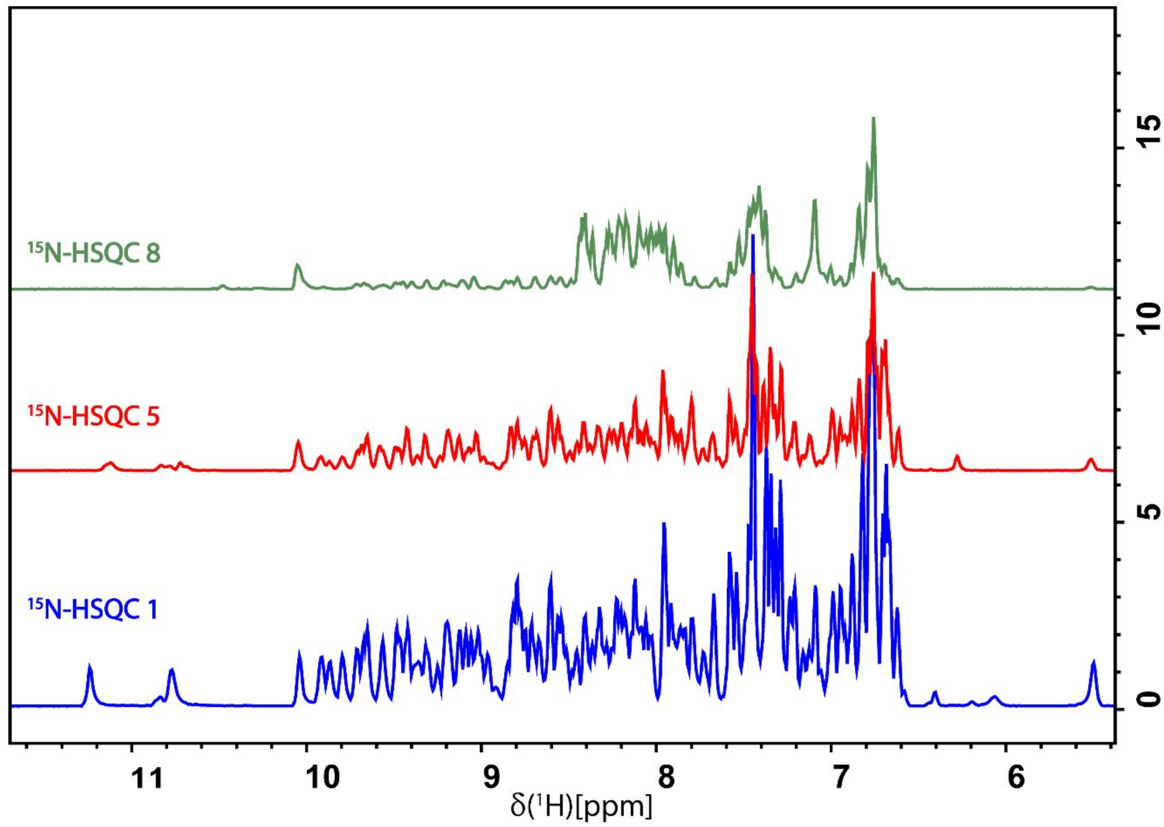

Figure S12: ^{1}H projections from the 2D ^{15}N-HSQC spectra of *apo-Sm*LPMO10A (^{15}N-HSQC 1), *holo-Sm*LPMO10A incubated with 10 mM ascorbic acid for t= 40h in the absence of the substrate chitin (^{15}N-HSQC 5), and *holo-Sm*LPMO10A, subsequently incubated with 5 mM ascorbic acid and 2 mM H_2O_2 for t'= 44h (^{15}N-HSQC 8).

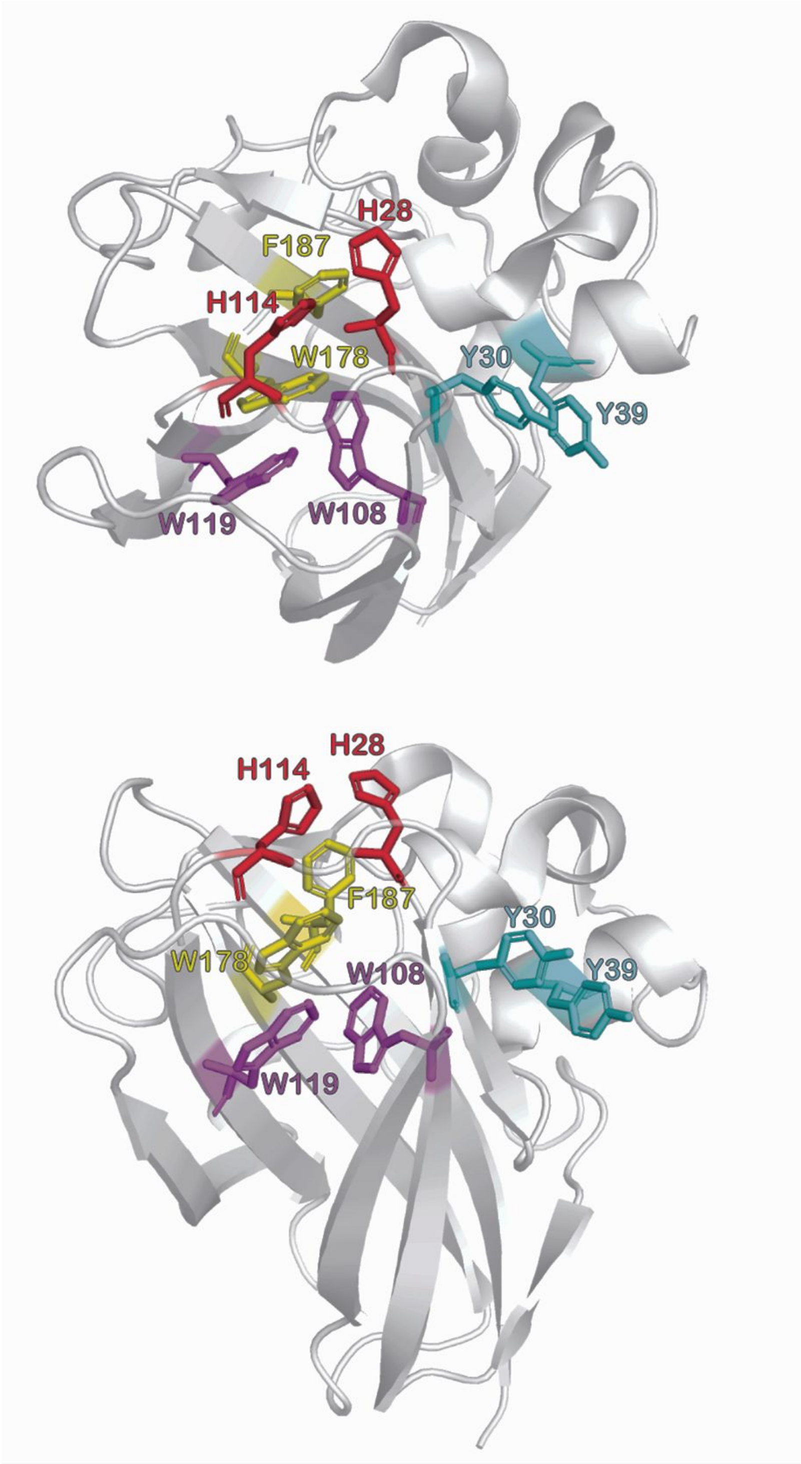

Figure S13: Aromatic pairs likely to give rise to π-π* excitation coupling in the CD spectra of SmLPMO10A. The three aromatic pairs Y30-Y39 (cyan), W108-W119 (magenta), and W178-F187 (yellow) are positioned in a way that allows for π-π* excitation coupling. In addition, π-π* excitation coupling is possible between residues W119 and W178 or F187. The aromatic residues are shown on a ribbon diagram of *Sm*LPMO10A (PDB ID: 2BEM).

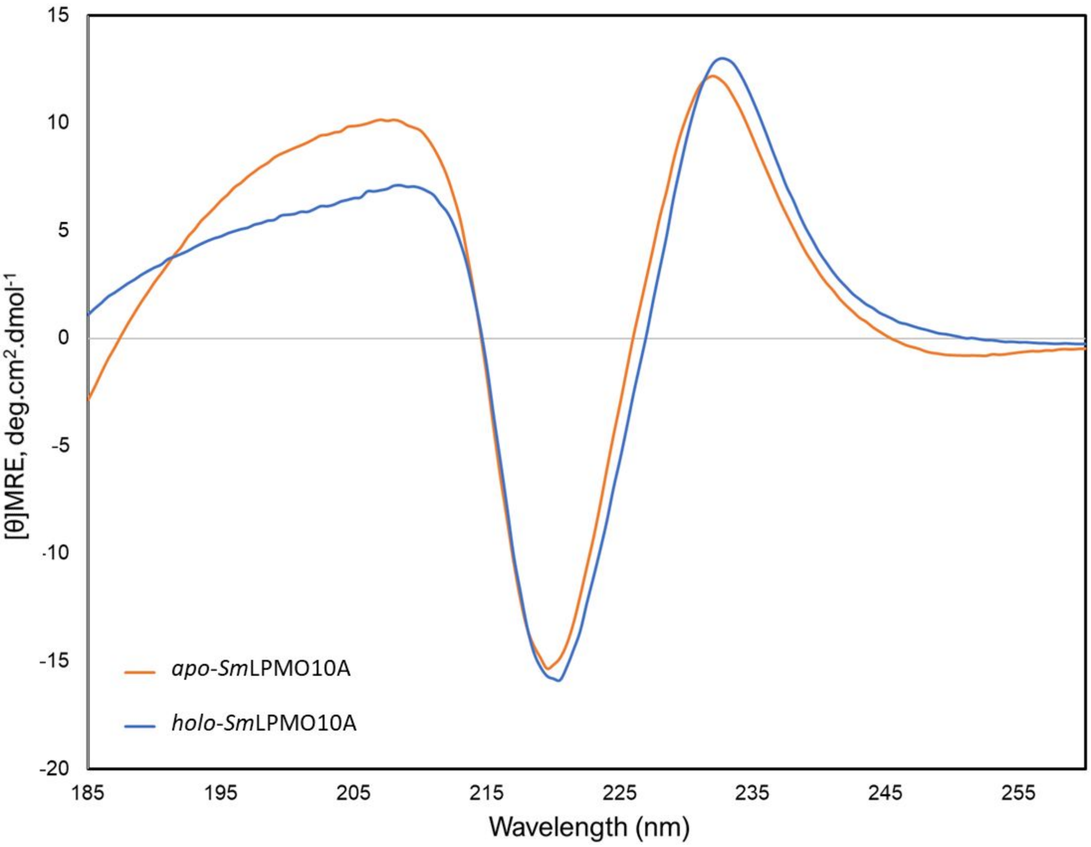

Figure S14: Far UV CD spectra of *apo*-*Sm*LPMO10A and *holo*-SmLPMO10A measured 23 °C. The protein concentration was 2.5 µM in all cases, and the protein was dissolved in acetate buffer (25 mM sodium-acetate, 10 mM NaCl, pH 5.0).

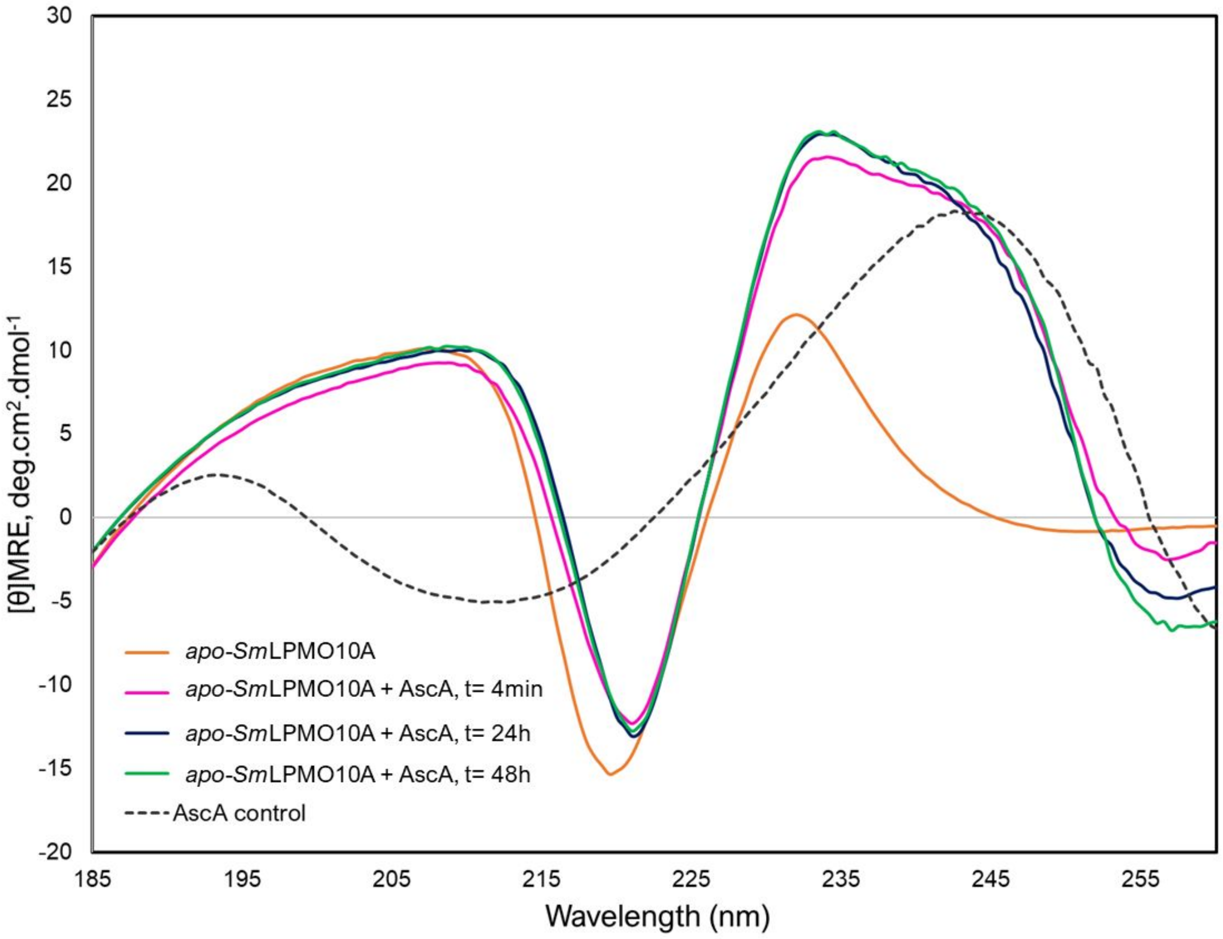

Figure S15: Far UV CD spectra of *apo*-*Sm*LPMO10A and *apo*-*Sm*LPMO10A incubated with 10 mM ascorbic acid for 4min, 24h, or 48h in the absence of the substrate chitin at 23 °C. The protein concentration was 2.5 µM in all cases and the protein was dissolved in acetate buffer (25 mM sodium-acetate, 10 mM NaCl, pH 5.0).

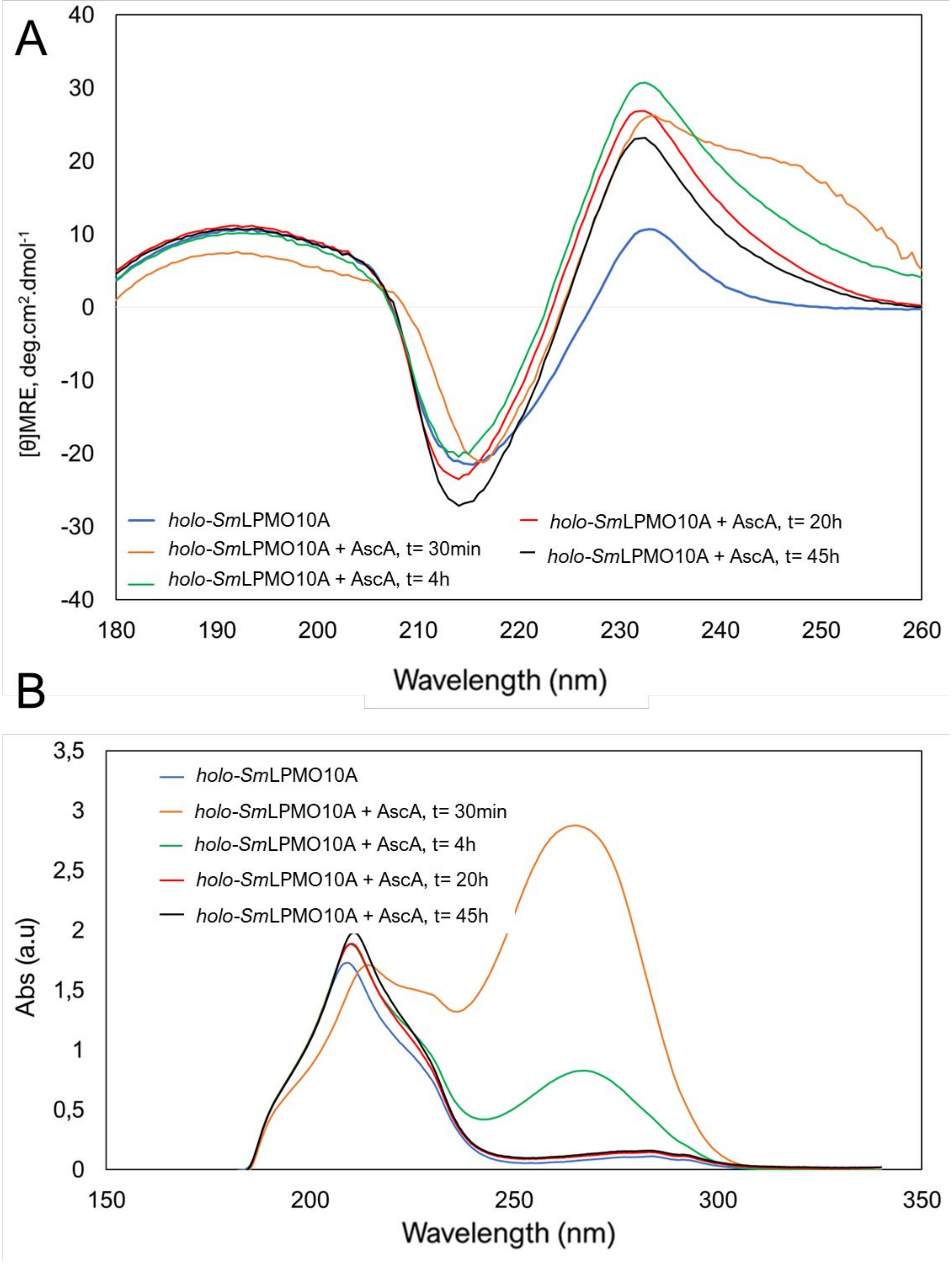

Figure S16: Far UV CD (A) and UV absorbance (B) spectra of *holo*-*Sm*LPMO10A and *holo*-SmLPMO10A incubated with 10 mM ascorbic acid for 30min, 4h, 20h, or 45h in the absence of chitin at 23 °C. The protein concentration was 2.5 µM in all cases and the protein was dissolved in acetate buffer (25 mM sodium-acetate, 10 mM NaCl, pH 5.0).

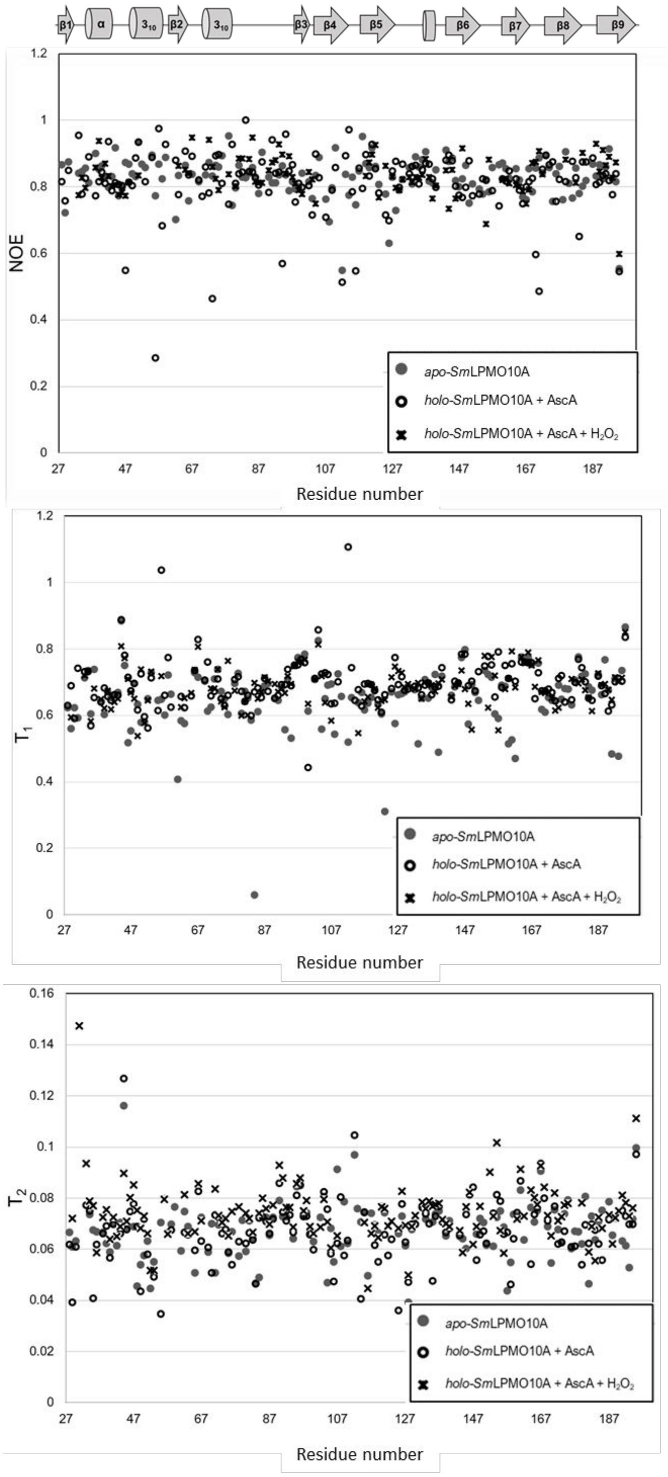

Figure S17: Backbone dynamics of *apo*-*Sm*LPMO10A, *holo*-*Sm*LPMO10A incubated with 10 µM ascorbic acid for t= 96h, and *holo*-*Sm*LPMO10A incubated with an additional 5 mM ascorbic acid and 2 mM H_2O_2 for t= 72h. Lower {^{1}H}-^{15}N NOE, T_1 and T2 values indicate greater protein backbone flexibility. However, clear changes in the structure of *Sm*LPMO10A in response to incubation with ascorbic acid and H_2O_2 would be seen in the same areas of the structure in all three experiments. No such conformational changes were clear in the displayed results which were ambiguous.

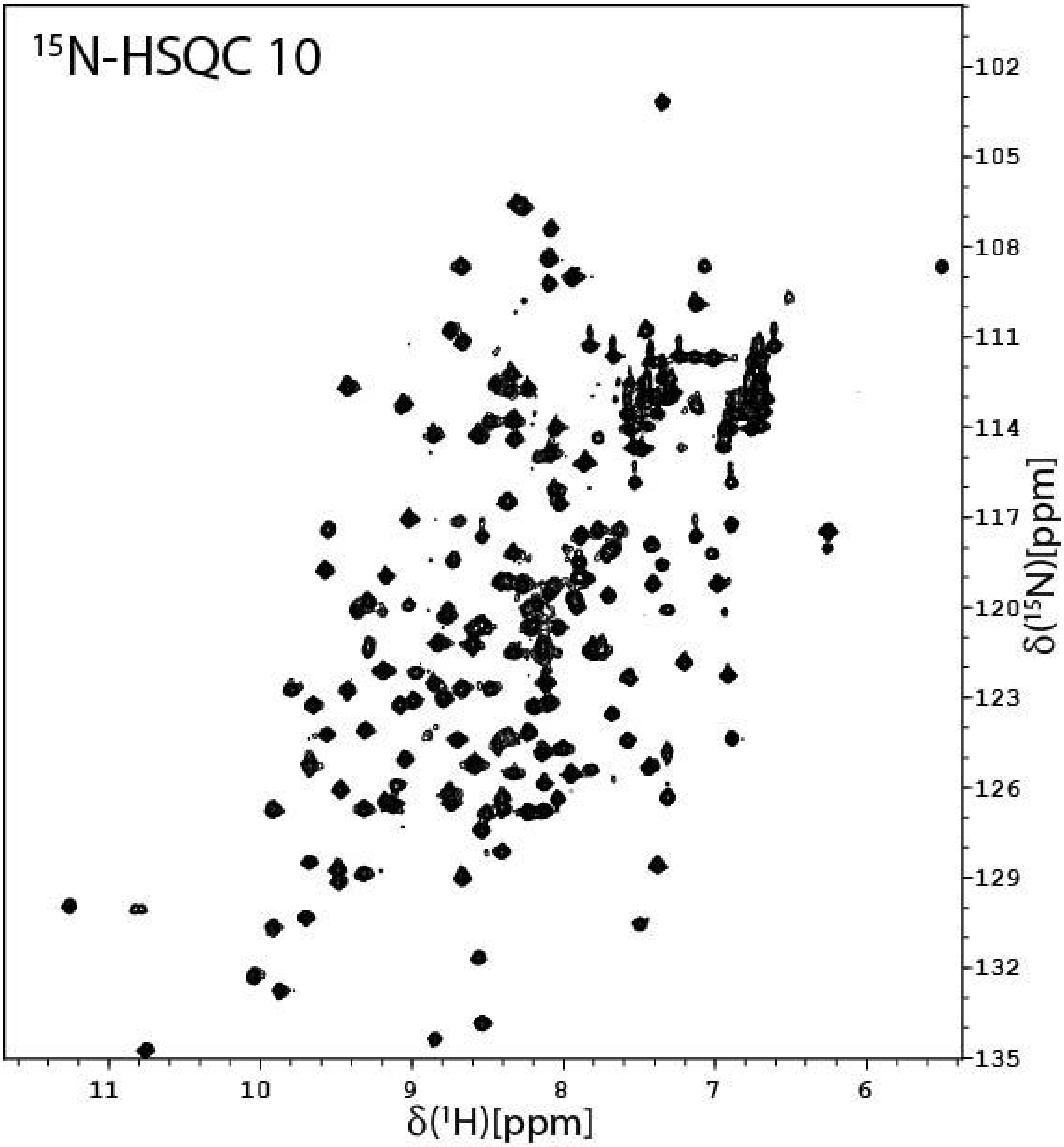

Figure S18: The ^{15}N-HSQC spectrum of *holo-Sm*LPMO10A incubated with 10 mM ascorbic acid for t= 0h in acetate buffer (25 mM sodium-acetate, 10 mM NaCl, pH 5.0) in the presence of 10 mg β-chitin particles with a size of ~ 0.5 mm (1 h preincubation with chitin prior to addition of ascorbic acid) at 25 °C (treatment series 2; ^{15}N-HSQC 10).

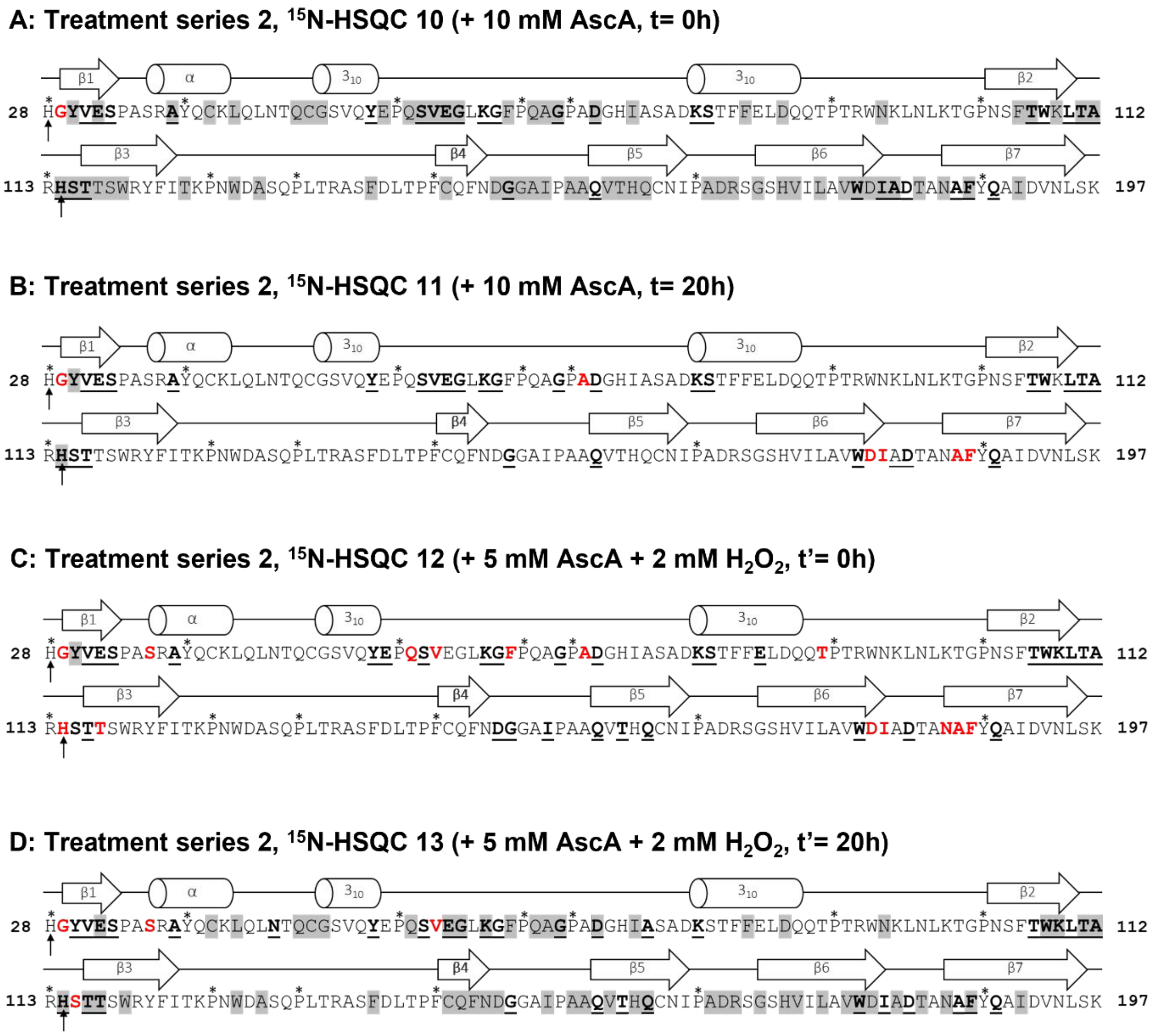

Figure S19: The combined structural changes to *holo*-*Sm*LPMO10A observed in treatment series 2 in the presence of the substrate β-chitin (1h pre-incubation), relative to the reference spectrum of *apo*-*Sm*LPMO10A. The active site histedines are indicated by arrows, while residues not assigned in by NMR are indicated by asterixis. Chemical shift perturbations >50Hz are shown as by bold and underlined residues, narrower linewidths (i.e increased signal intensities) are shown by residues highlighted in grey. Non-detectable ^{1}H-^{15}N signals are shown by red residues.

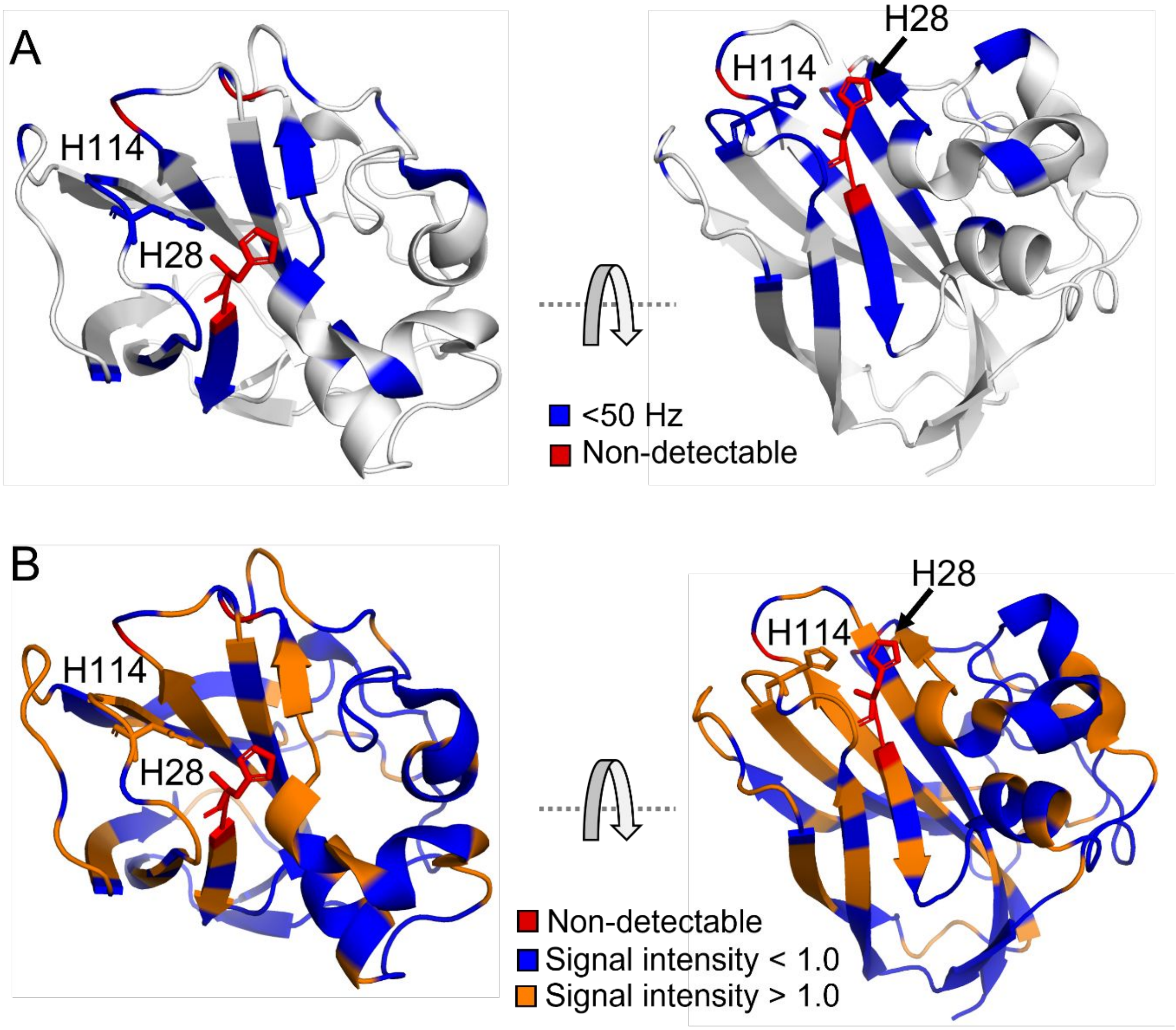

Figure S20: Immediate (t= 0h) effect of incubating *holo*-*Sm*LPMO10A with 10 mM ascorbic acid under aerobic conditions in the presence of β-chitin particles (treatment series 2). **A)** Chemical Shift Perturbations (CSPs) in response to addition of ascorbic acid (at t= 0h) and copper in the presence of chitin compared to *apo*-*Sm*LPMO10A highlighted on the X-ray crystal structure of *Sm*LPMO10A (PDB ID: 2BEM). The magnitude of the change in Hz is shown by the color scheme. Non-detectable residues are shown in red. **B)** Narrower linewidths in response to addition of ascorbic acid (at t= 0h) and copper reduction in the presence of chitin compared with *apo*-*Sm*LPMO10A highlighted on the X-ray crystal structure of *Sm*LPMO10A. An increase in signal intensity is shown in orange, while a decrease is shown in blue. Non-detectable residues are shown in red.

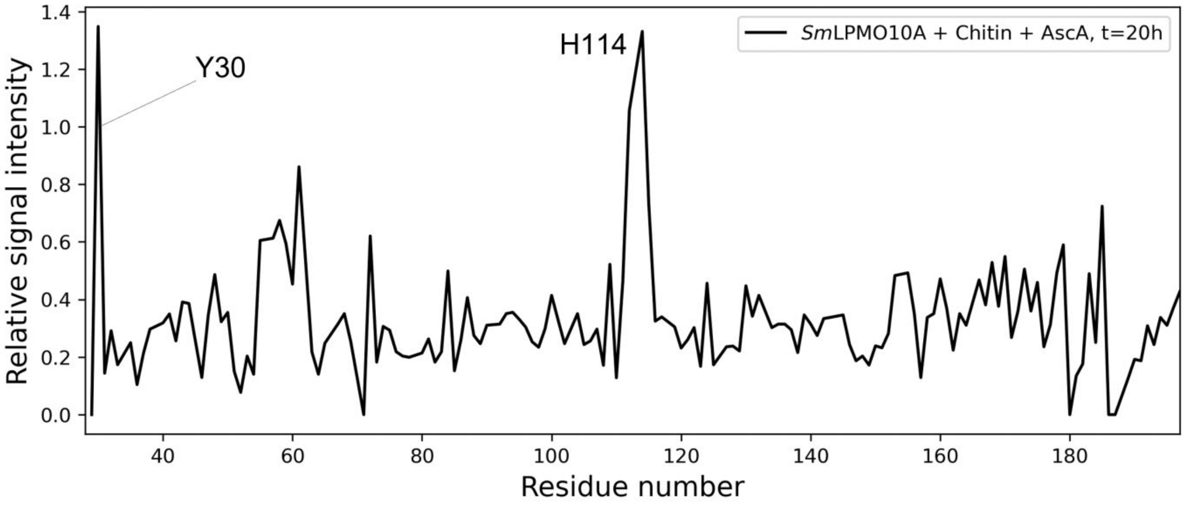

Figure S21: The relative intensities of ^{1}H-^{15}N signals in the ^{15}N-HSQC spectra of *holo*-*Sm*LPMO10A incubated with 10 mM ascorbic acid for t= 20h in the presence of chitin (^{15}N-HSQC 11) and the intensities of ^{1}H-^{15}N signal intensities in the ^{15}N-HSQC spectrum of *apo*-*Sm*LPMO10A in the absence of chitin (^{15}N-HSQC 1). Only Y30 and H114 show an increase in signal intensity.

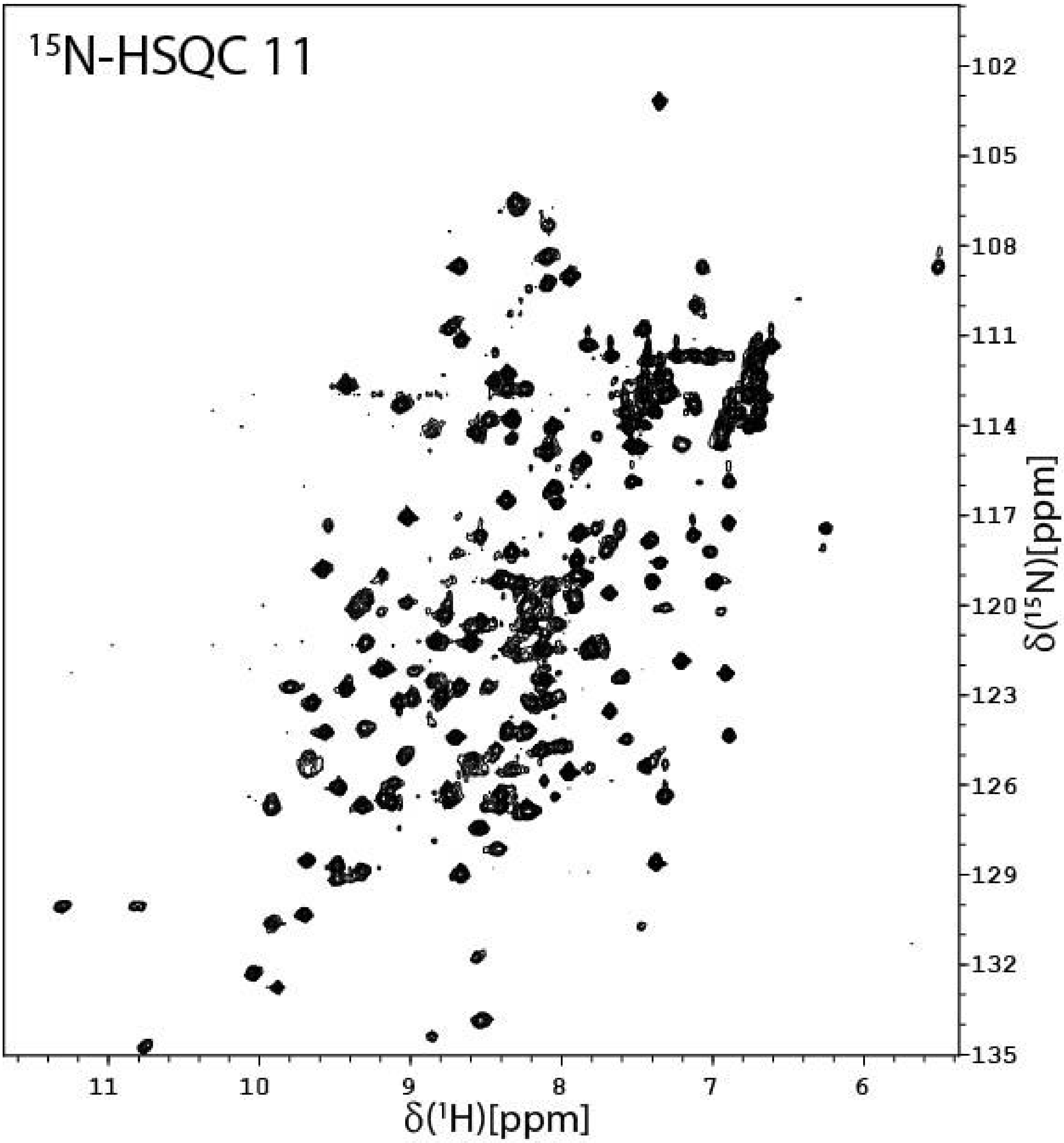

Figure S22: The ^{15}N-HSQC of *holo*-*Sm*LPMO10A incubated with 10 mM ascorbic acid for t= 20h in acetate buffer (25 mM sodium-acetate, 10 mM NaCl, pH 5.0) in the presence of 10 mg β-chitin particles with a size of ~ 0.5 mm (1h preincubation) at 25 °C (treatment series 2, ^{15}N-HSQC 11).

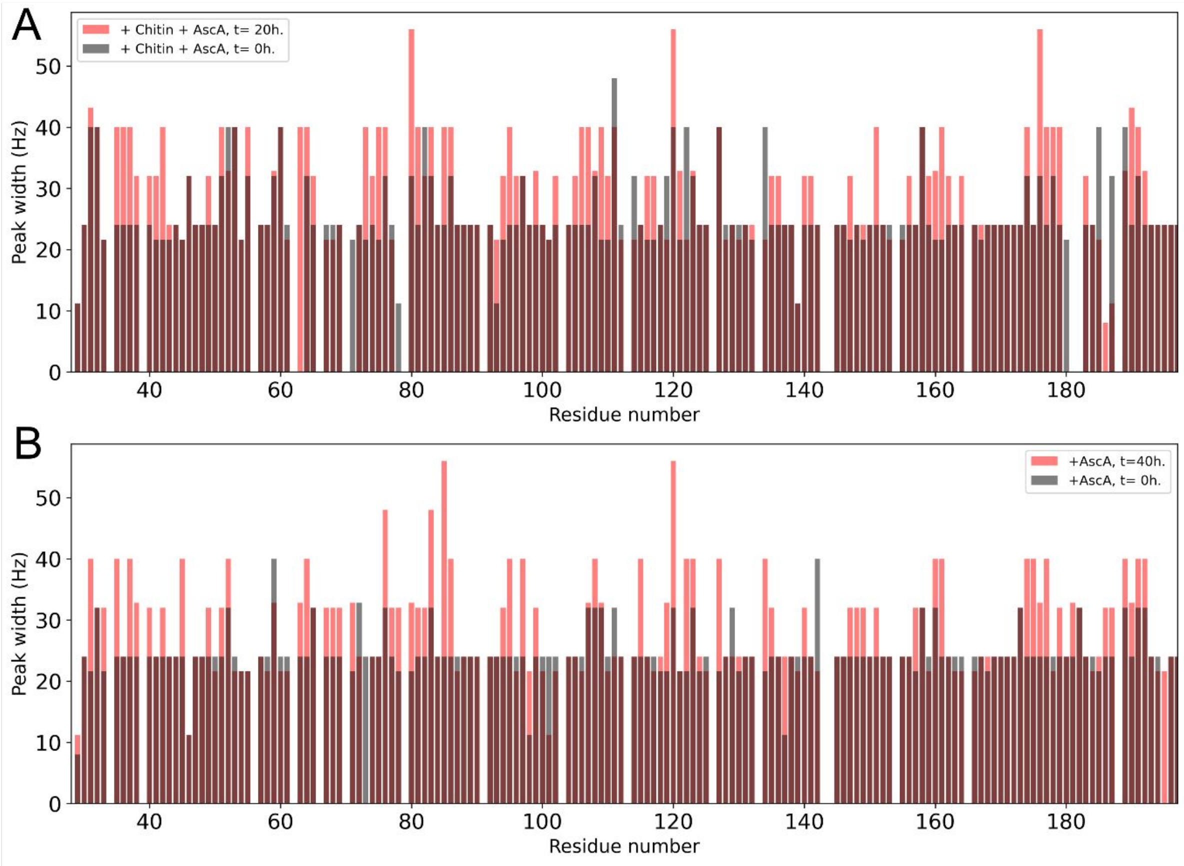

Figure S23: A) Changes in the ^{1}H resonance linewidth of all residues of *holo*-*Sm*LPMO10A in response to t= 0h (treatment series 2, ^{15}N-HSQC 10) and t= 20h (treatment series 2, ^{15}N-HSQC 11) incubation periods with ascorbic acid in the presence of chitin (1h pre-incubation with chitin alone). **B)** Changes in the ^{1}H resonance linewidth of all residues of *holo*-*Sm*LPMO10A in response to t= 0h (treatment series 1, ^{15}N-HSQC 3) and t= 40h (treatment series 1, ^{15}N-HSQC 5) incubation periods with ascorbic acid.

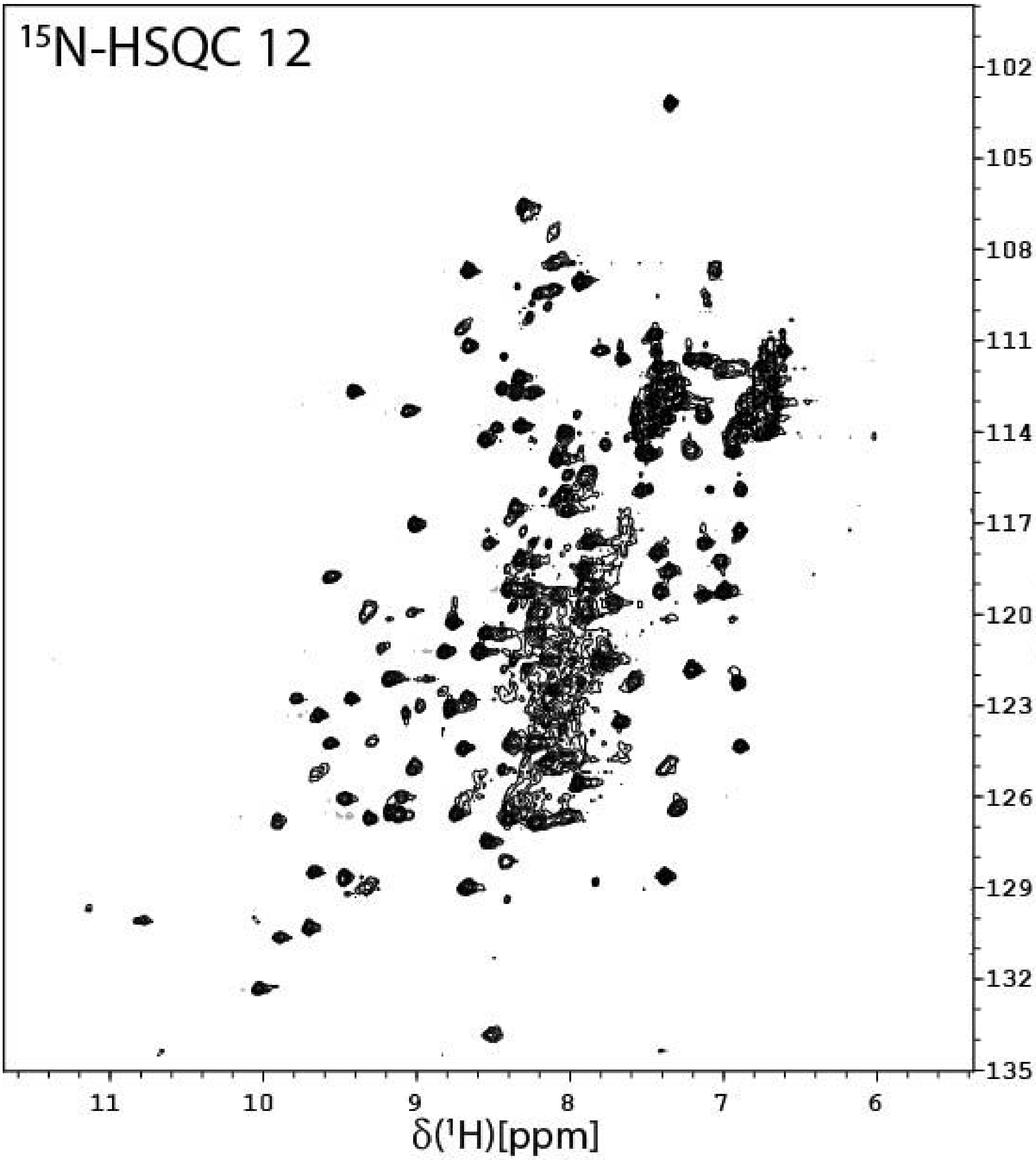

Figure S24: The ^{15}N-HSQC of *holo*-*Sm*LPMO10A incubated with 10 mM ascorbic acid for 20h and, subsequently, with 5 mM ascorbic acid and 2 mM H_2O_2 for t'= 0h in acetate buffer (25 mM sodium-acetate, 10 mM NaCl, pH 5.0) in the presence of 10 mg β-chitin particles with a size of ~ 0.5 mm (1h preincubation) at 25 °C (treatment series 2, ^{15}N-HSQC 12).

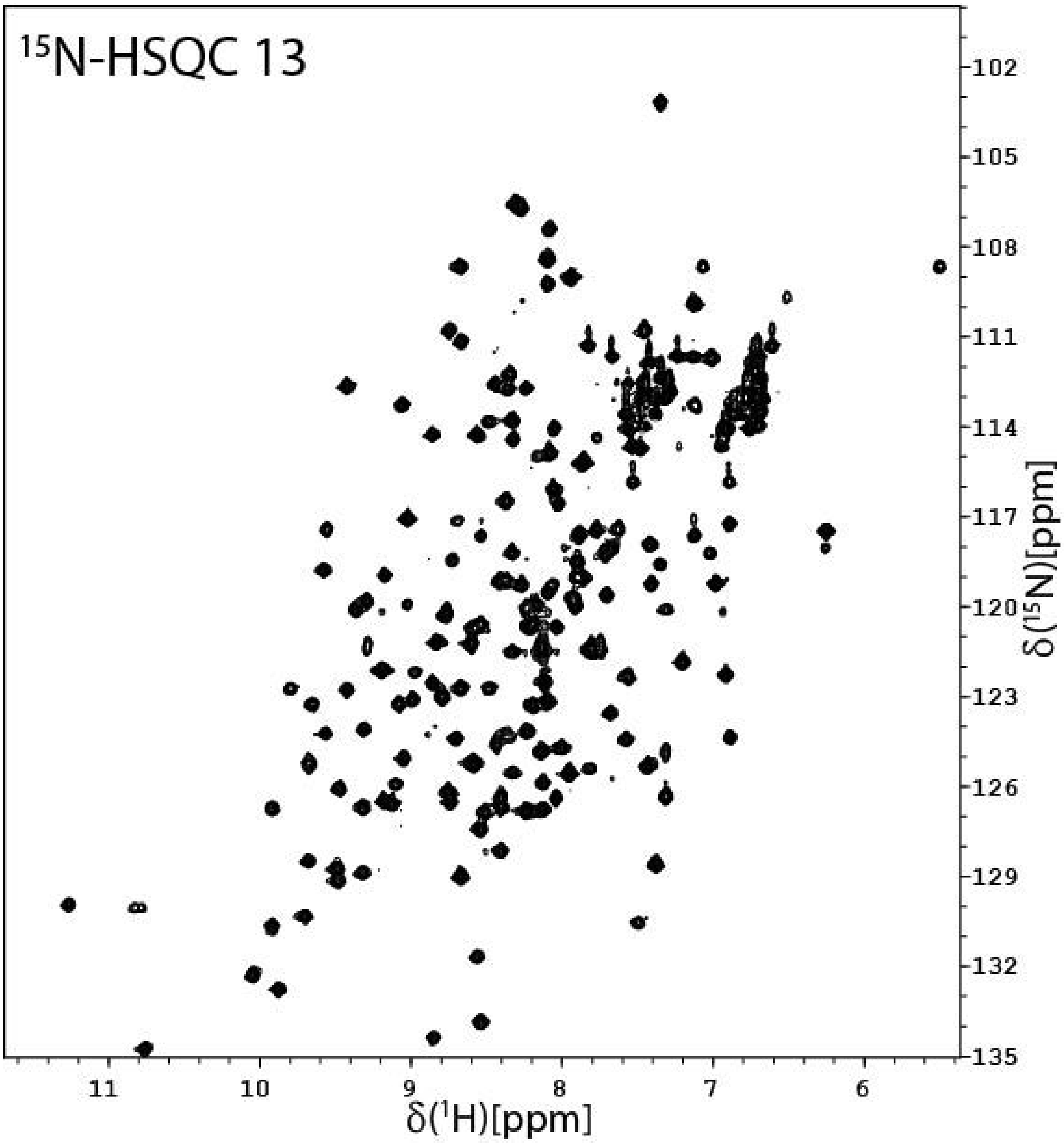

Figure S25: The ^{15}N-HSQC of *holo*-*Sm*LPMO10A incubated with 10 mM ascorbic acid for 20h and, subsequently, with 5 mM ascorbic acid and 2 mM H_2O_2 for t'= 20h in acetate buffer (25 mM sodium-acetate, 10 mM NaCl, pH 5.0) in the presence of 10 mg β-chitin particles with a size of ~ 0.5 mm (1h preincubation) at 25 °C (treatment series 2, ^{15}N-HSQC 13).

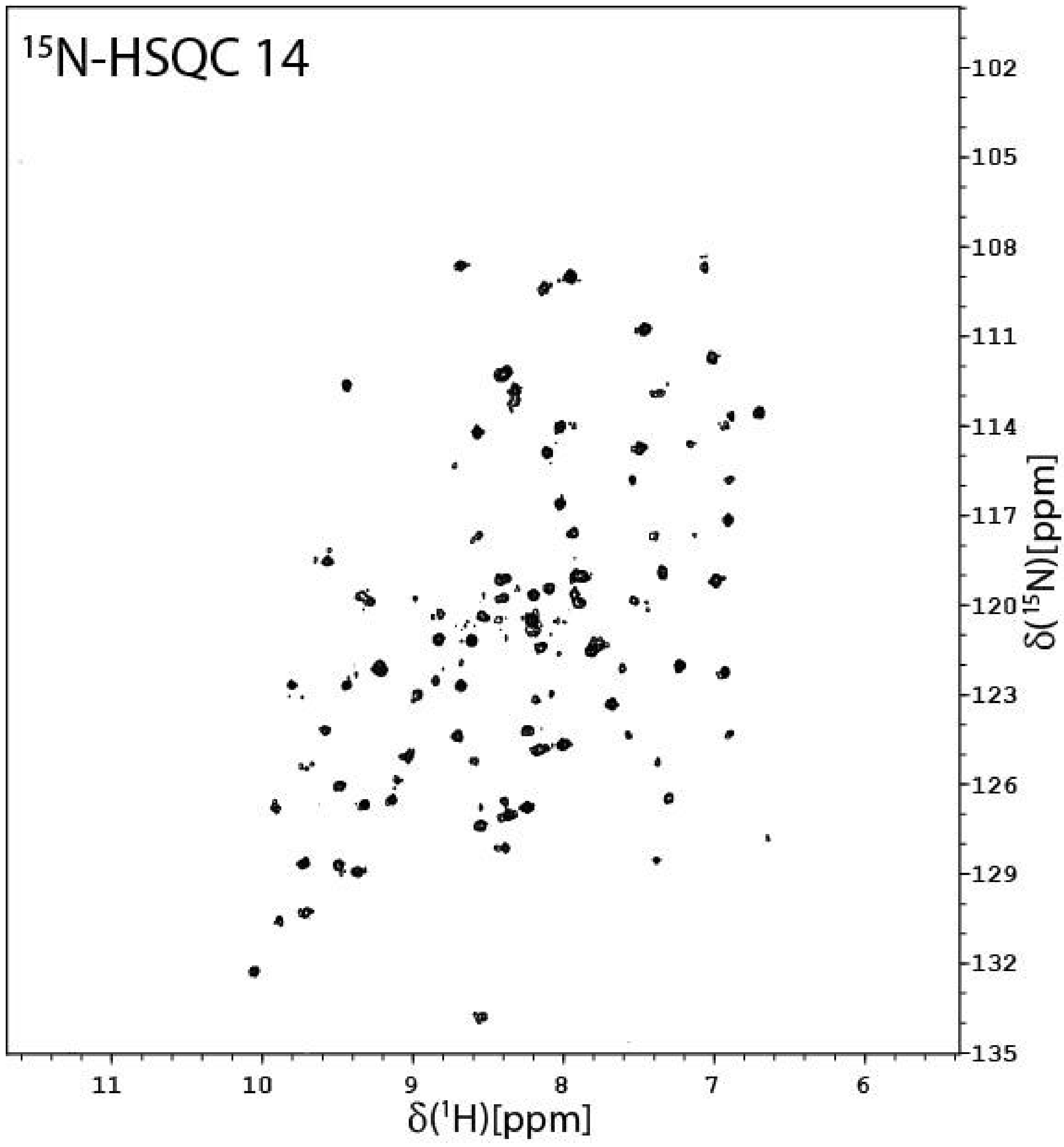

Figure S26: The ^{15}N-HSQC of *holo*-*Sm*LPMO10A in acetate buffer (25 mM sodium-acetate, 10 mM NaCl, pH 5.0) in the presence of 10 mg β-chitin particles with a size of ~ 0.5 mm (24h preincubation) at 25 °C (treatment series 3, 15N-HSQC 14).

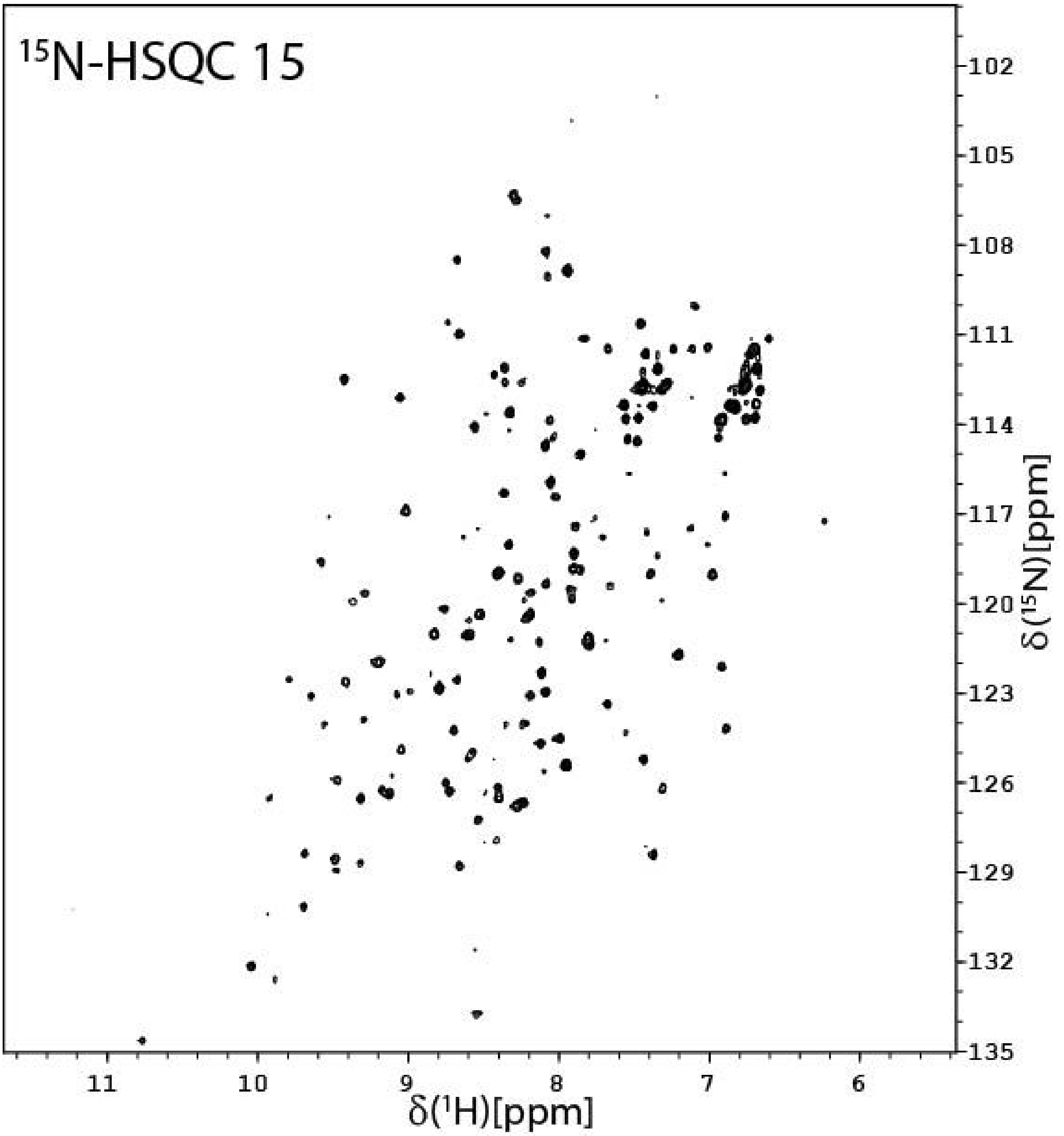

Figure S27: The ^{15}N-HSQC of *holo*-*Sm*LPMO10A incubated with 10 mM ascorbic acid for t= 0h in acetate buffer (25 mM sodium-acetate, 10 mM NaCl, pH 5.0) in the presence of 10 mg β-chitin particles with a size of ~ 0.5 mm (24h preincubation) at 25 °C (treatment series 3, ^{15}N-HSQC 15).

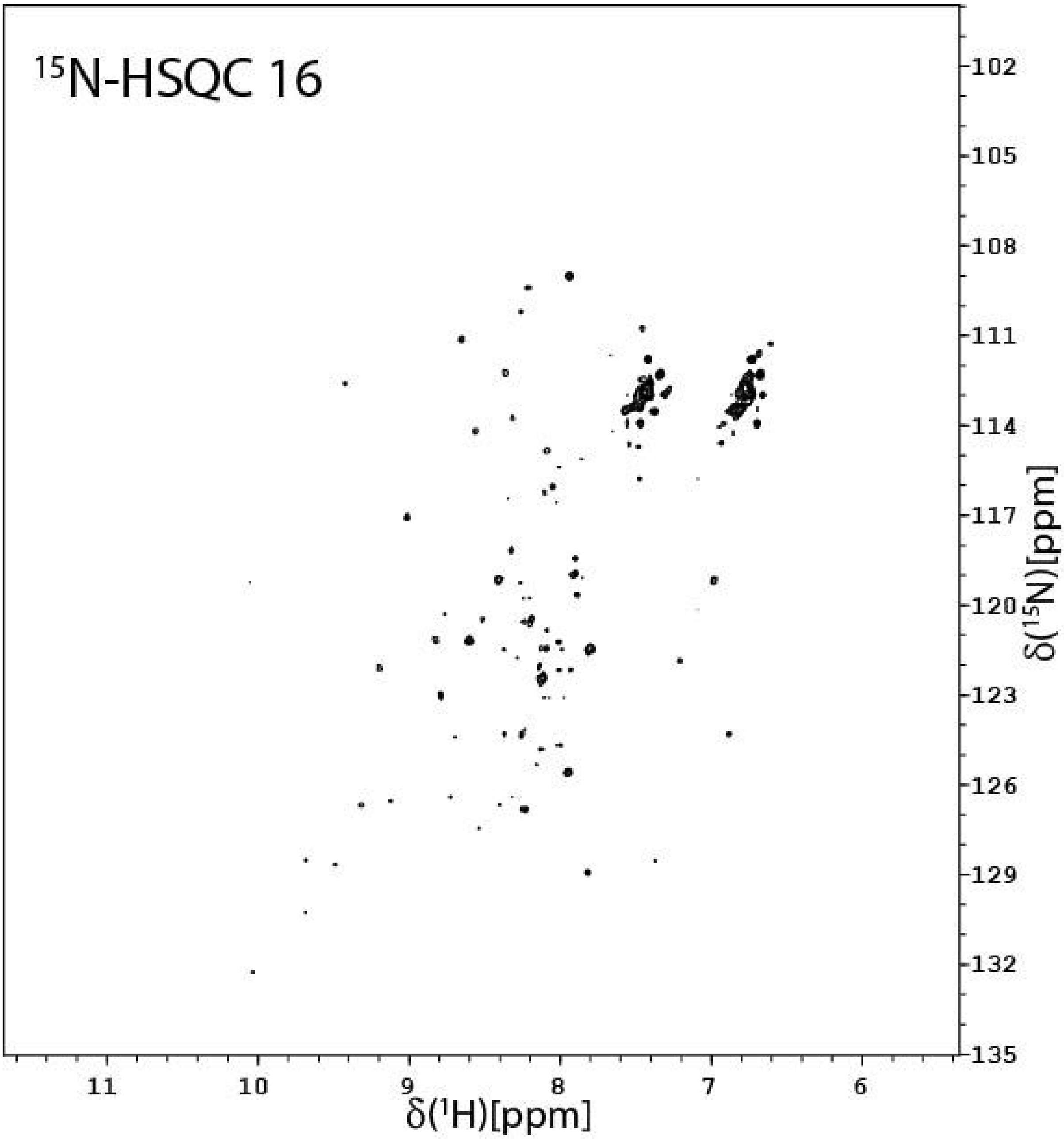

Figure S28: The ^{15}N-HSQC of *holo*-*Sm*LPMO10A incubated with 10 mM ascorbic acid for t= 24h in acetate buffer (25 mM sodium-acetate, 10 mM NaCl, pH 5.0) in the presence of 10 mg β-chitin particles with a size of ~ 0.5 mm (24h preincubation) at 25 °C (treatment series 3, ^{15}N-HSQC 16).

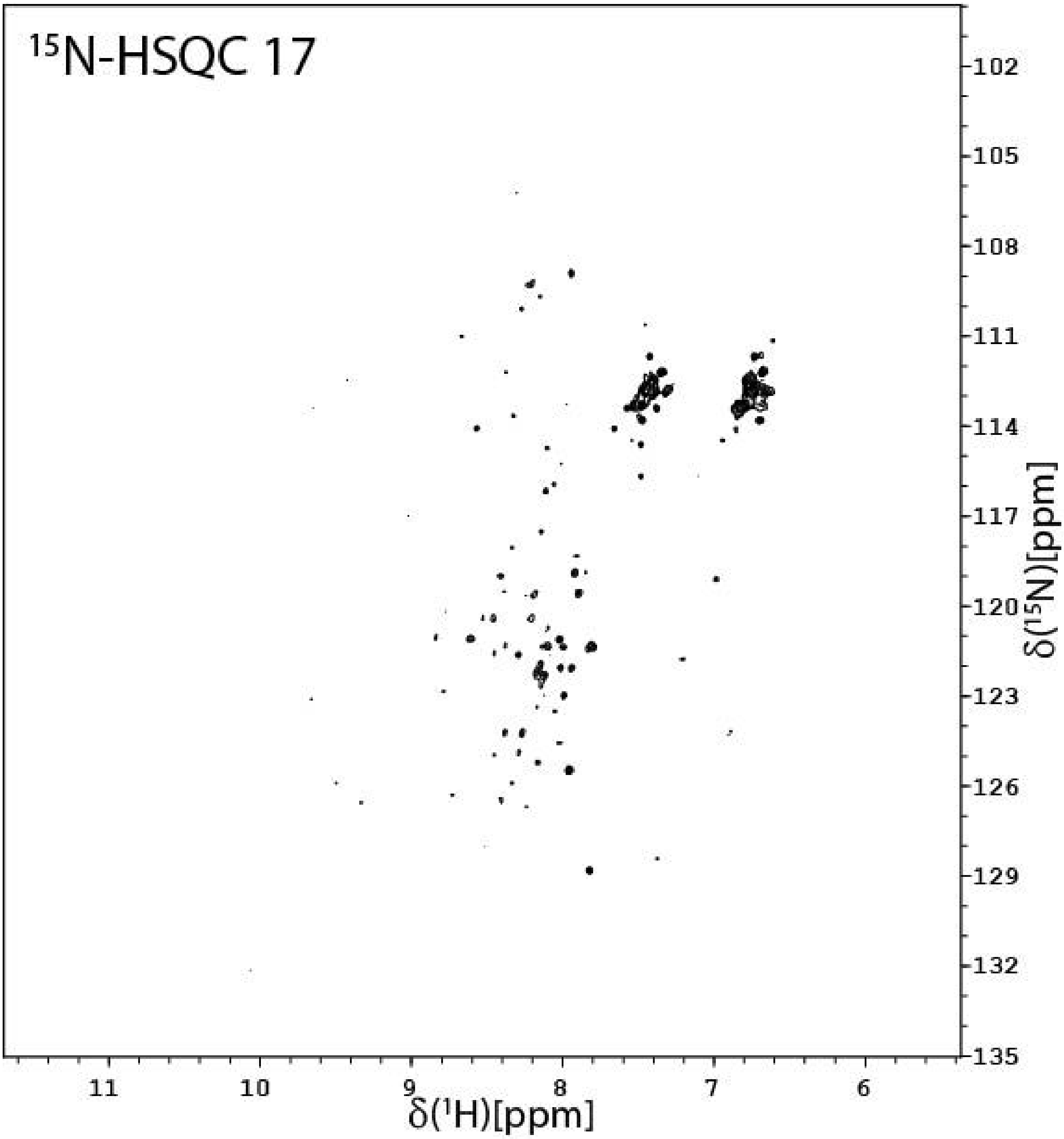

Figure S29: The ^{15}N-HSQC of *holo*-*Sm*LPMO10A incubated with 10 mM ascorbic acid for t= 48h in acetate buffer (25 mM sodium-acetate, 10 mM NaCl, pH 5.0) in the presence of 10 mg β-chitin particles with a size of ~ 0.5 mm (24h preincubation) at 25 °C (treatment series 3, ^{15}N-HSQC 17).

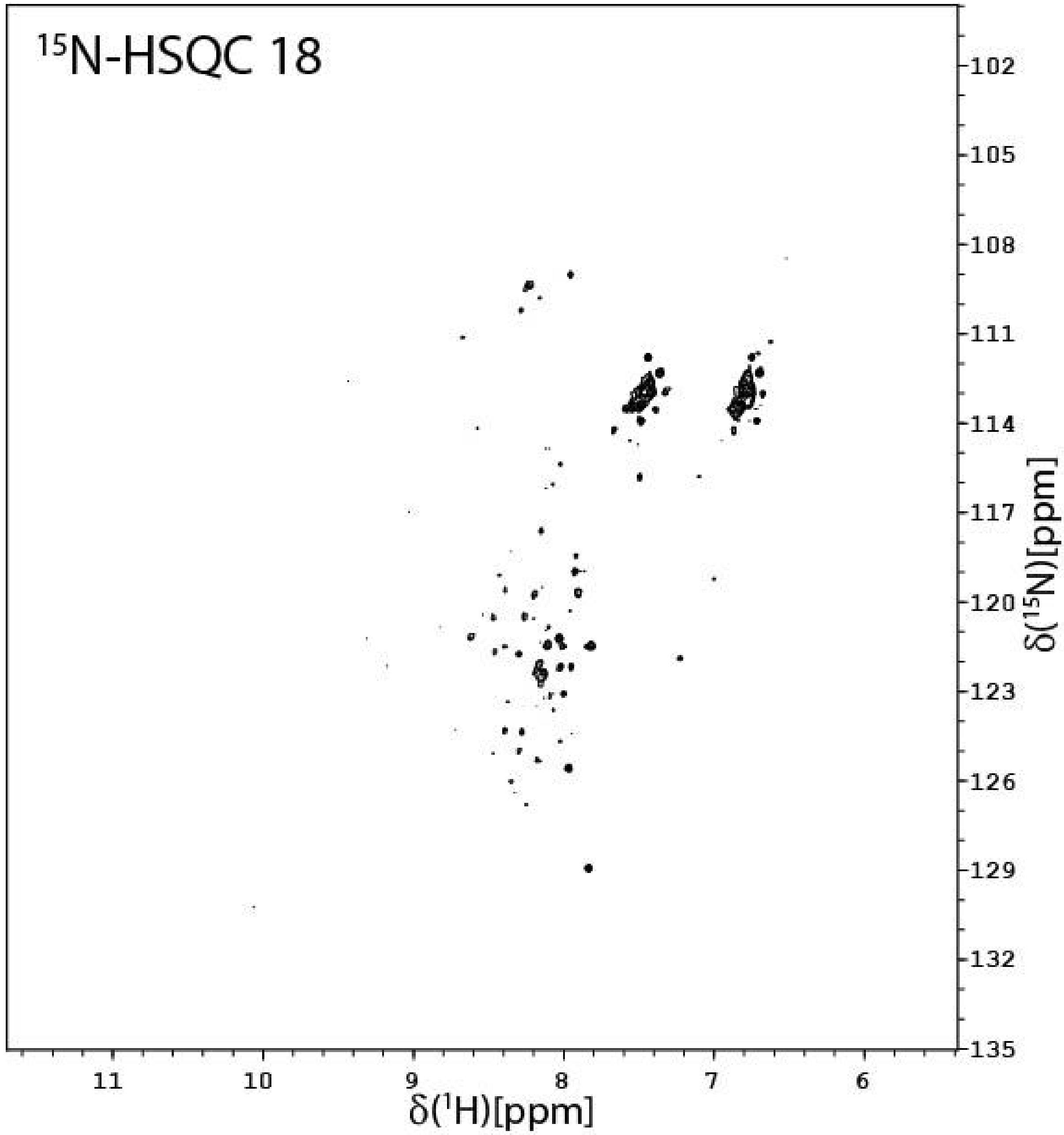

Figure S30: The ^{15}N-HSQC of *holo*-*Sm*LPMO10A incubated with 10 mM ascorbic acid for t= 78h in acetate buffer (25 mM sodium-acetate, 10 mM NaCl, pH 5.0) in the presence of 10 mg β-chitin particles with a size of ~ 0.5 mm (24h preincubation) at 25 °C (treatment series 3, ^{15}N-HSQC 18).

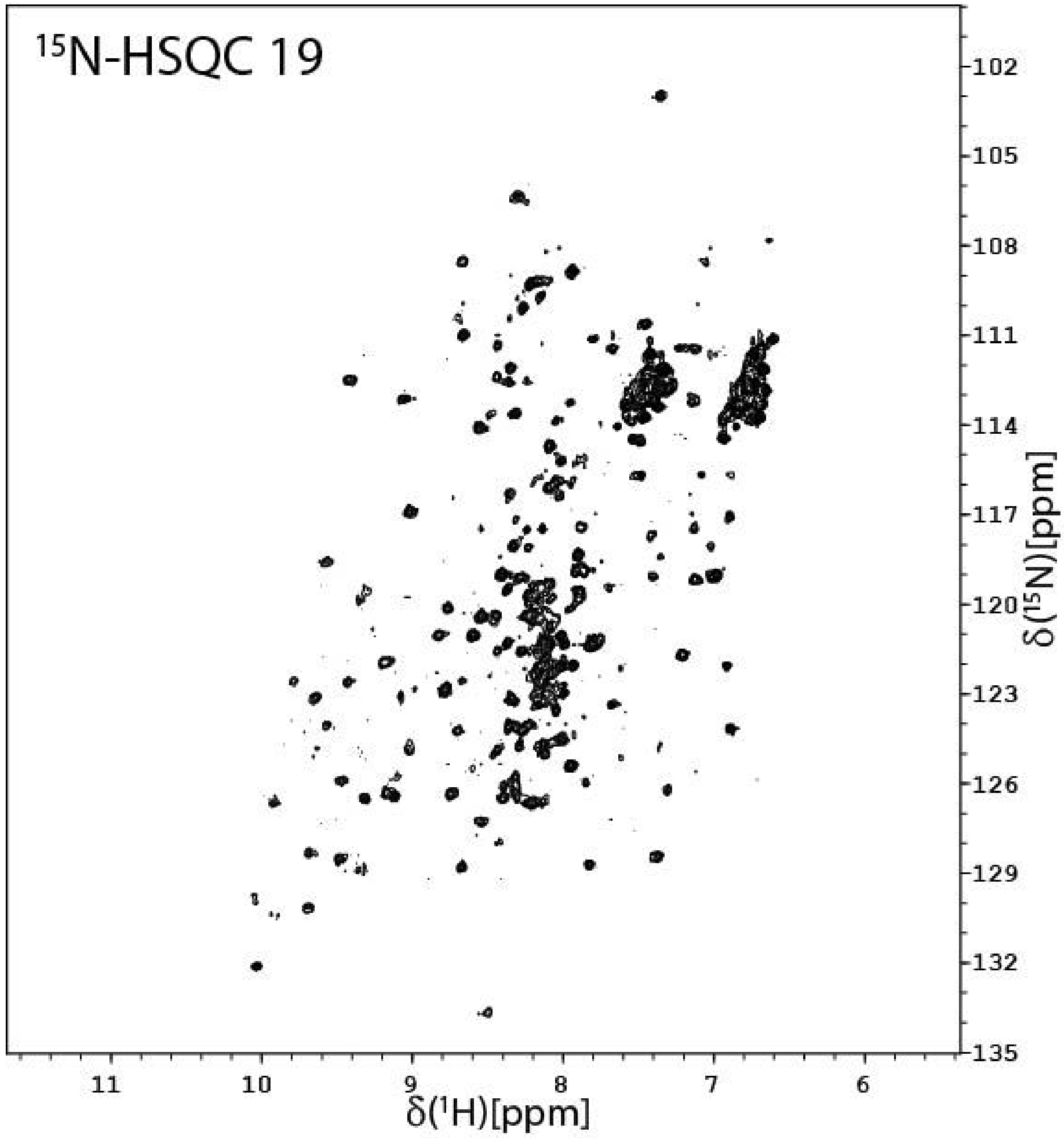

Figure S31: The ^{15}N-HSQC of *holo*-*Sm*LPMO10A incubated with 10 mM ascorbic acid for 78h and then 5 mM ascorbic acid and 2 mM H_2O_2 for t'= 0h in acetate buffer (25 mM sodium-acetate, 10 mM NaCl, pH 5.0) in the presence of 10 mg β-chitin particles with a size of ~ 0.5 mm (24h preincubation) at 25 °C (treatment series 3, ^{15}N-HSQC 19).

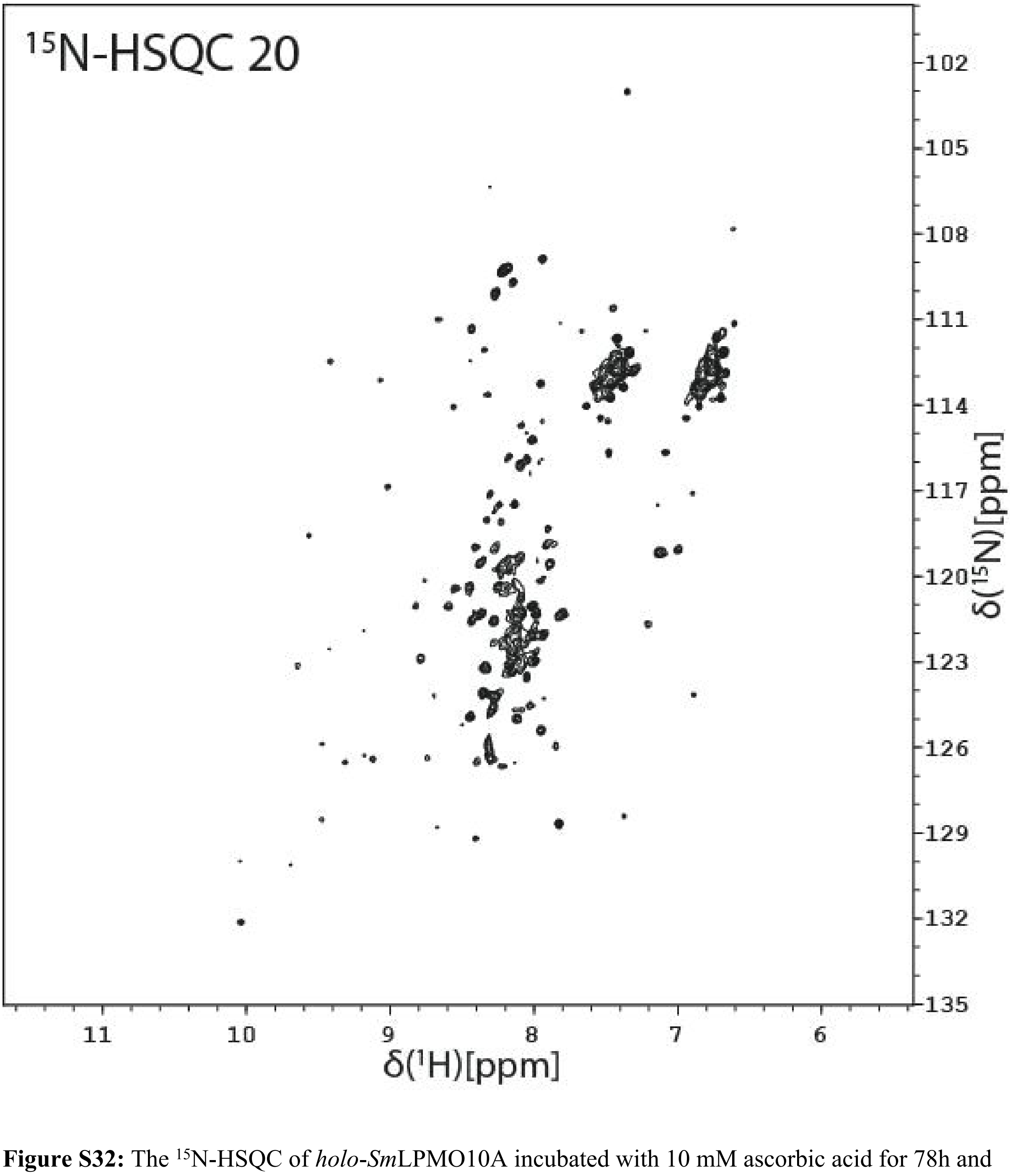

Figure S32: The ^{15}N-HSQC of *holo*-*Sm*LPMO10A incubated with 10 mM ascorbic acid for 78h and then 5 mM ascorbic acid and 2 mM H_2O_2 for t'= 24h in acetate buffer (25 mM sodium-acetate, 10 mM NaCl, pH 5.0) in the presence of 10 mg β-chitin particles with a size of ~ 0.5 mm (24h preincubation) at 25 °C (treatment series 3, ^{15}N-HSQC 20).

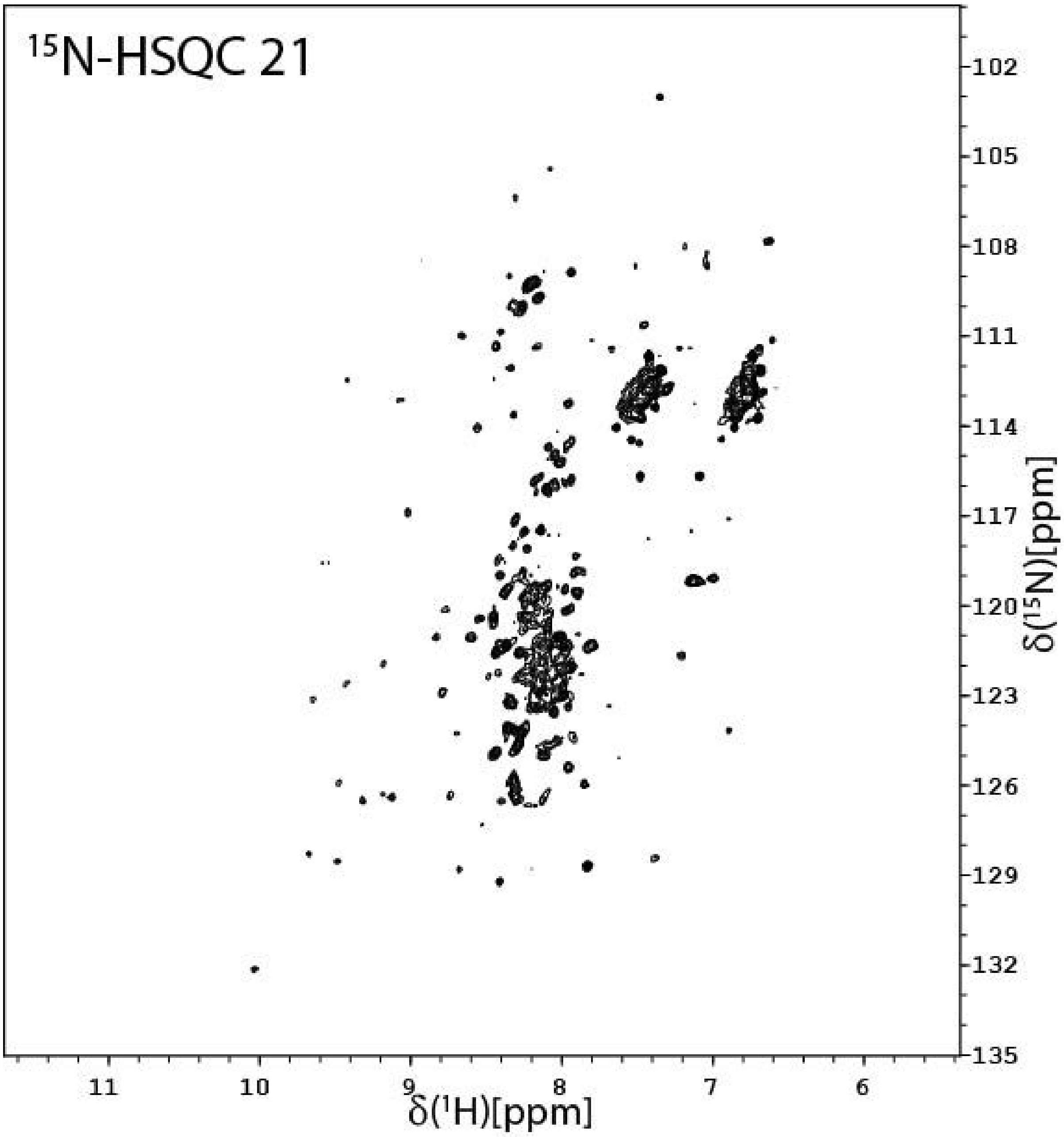

Figure S33: The ^{15}N-HSQC of *holo*-*Sm*LPMO10A incubated with 10 mM ascorbic acid for 78h and then 5 mM ascorbic acid and 2 mM H_2O_2 for t'= 48h in acetate buffer (25 mM sodium-acetate, 10 mM NaCl, pH 5.0) in the presence of 10 mg β-chitin particles with a size of ~ 0.5 mm (24h preincubation) at 25 °C (treatment series 3, ^{15}N-HSQC 21).

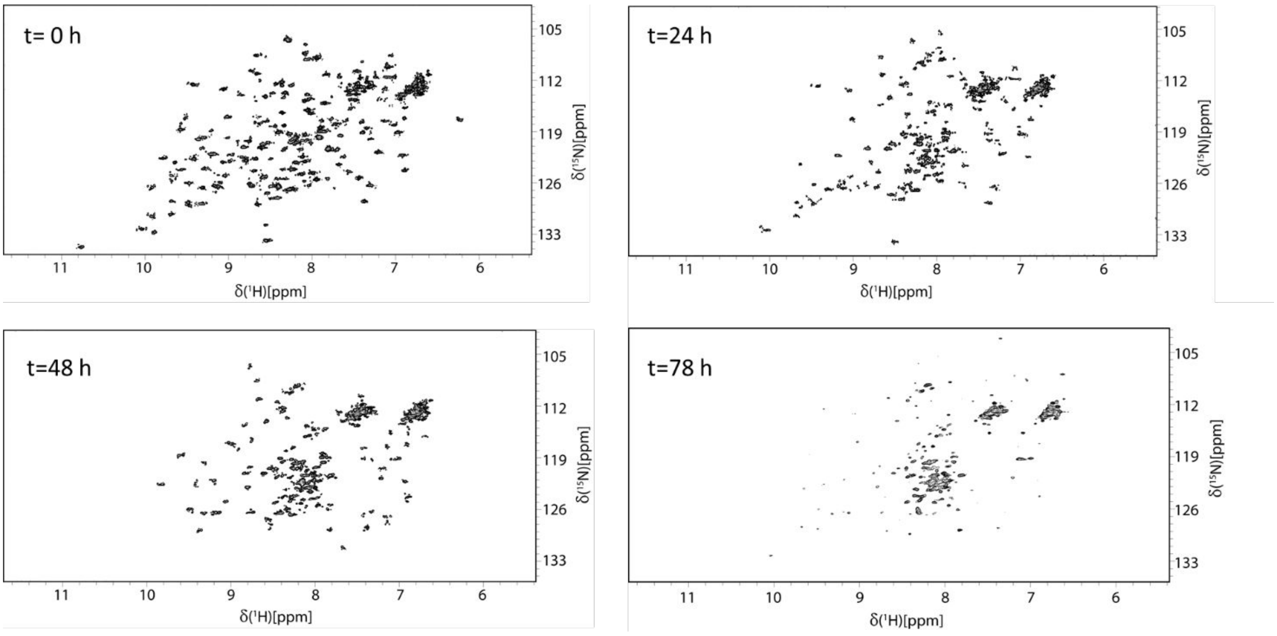

Figure S34: ^{15}N-HSQC spectra of *holo*-*Sm*LPMO10A (treatment series 3, ^{15}N-HSQCs 15-18) incubated with 10 mM ascorbic acid and 10 mg milled β-chitin for t= 0h, t= 24h, t= 48h, and t= 78h in acetate buffer (25 mM sodium-acetate, 10 mM NaCl, pH 5.0). Both reduced signal dispersion and signal line broadening, increased over time, indicating protein degradation. The enzyme was pre-incubated with the β-chitin particles for 24h before the addition of ascorbic acid.

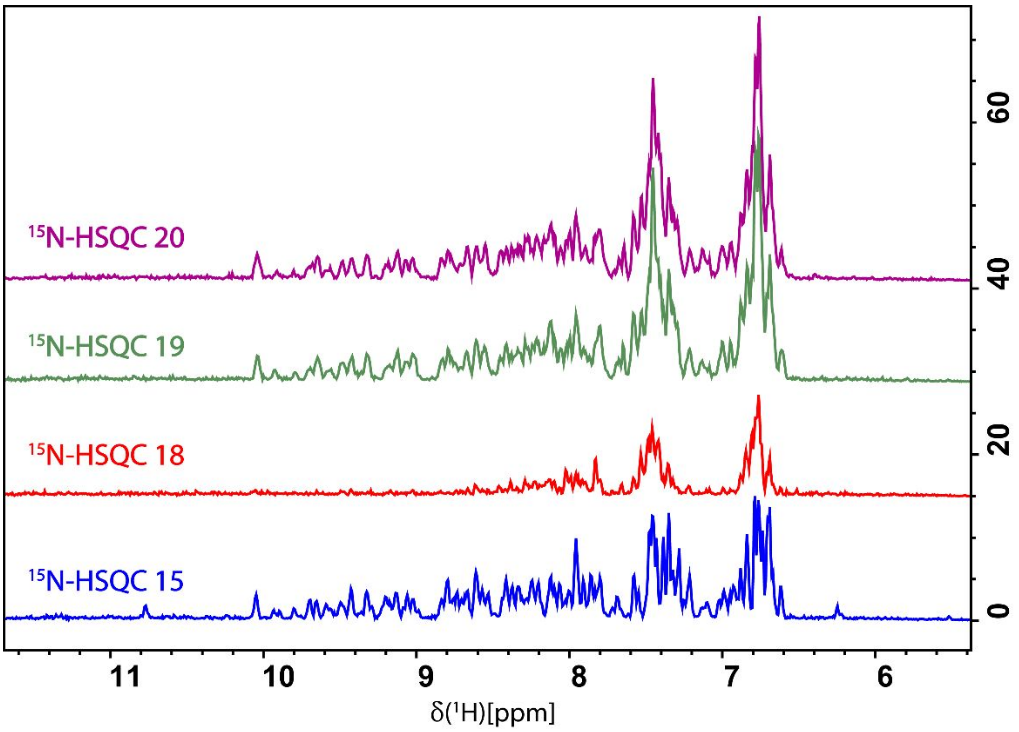

Figure S35: ^{1}H projections from the 2D ^{15}N-HSQC spectra of *holo*-*Sm*LPMO10A (pre-incubated with chitin for 24h in treatment series 3) incubated with 10 mM ascorbic acid for t= 0h (^{15}N-HSQC 15), t= 78h (^{15}N-HSQC 18), and, subsequently, 5 mM ascorbic acid + 2 mM H_2O_2 for t'= 0h (^{15}N-HSQC 19) and t'= 24h (^{15}N-HSQC 20).

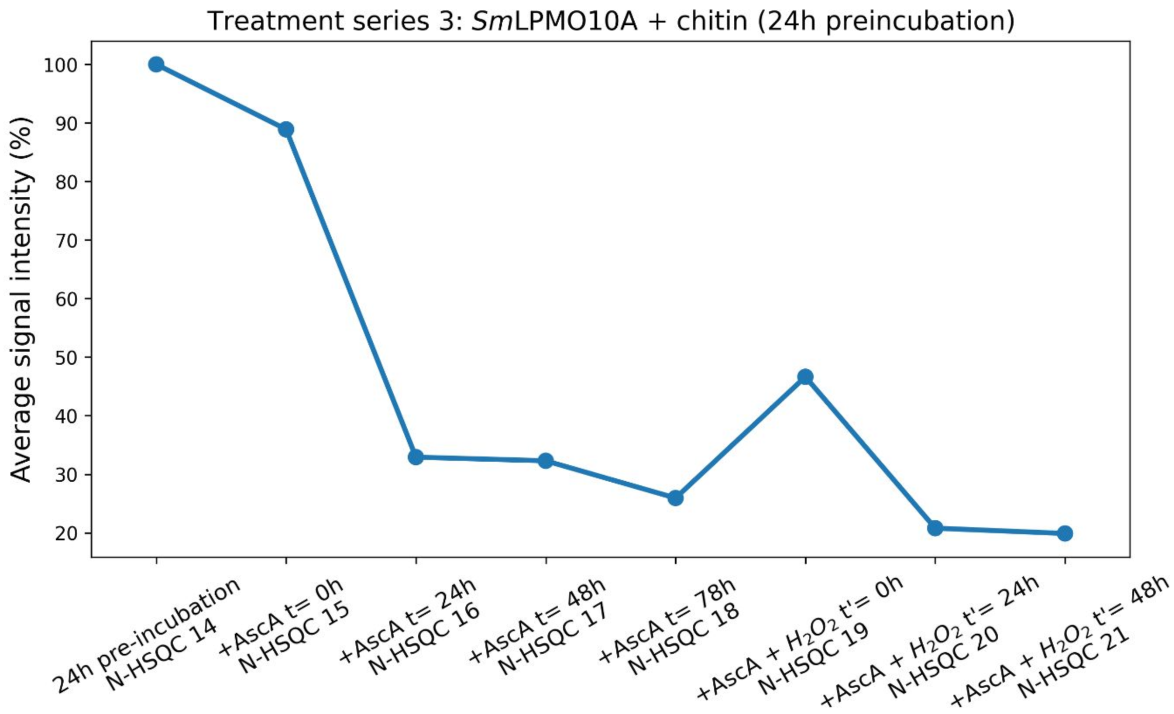

Figure S36: Average ^{1}H-signal intensities in ^{15}N-HSQC spectra 14-21 recorded for treatment series 3. *holo*-*Sm*LPMO10A was pre-incubated for 24 h with chitin, after which it was first exposed to 10 mM ascorbic acid alone for 78 h, and then to 5 mM ascorbic acid and 2 mM H_2O_2 for 48 h. The ^{1}H intensities of all the ^{1}H-^{15}N signals in ^{15}N-HSQC spectra 15-19 were integrated using CARA[1].

Literature:

(1) Keller, R. *The Computer Aided Resonance Assignment Tutorial*; CANTINA Verlag: Goldau, 2004.
